# An Automated Analysis of Homocoupling Defects Using MALDI-MS and Open-Source Computer Software

**DOI:** 10.1021/jasms.4c00225

**Published:** 2024-09-18

**Authors:** Maria Bochenek, Michał Aleksander Ciach, Sander Smeets, Omar Beckers, Jochen Vanderspikken, Błażej Miasojedow, Barbara Domżał, Dirk Valkenborg, Wouter Maes, Anna Gambin

**Affiliations:** † Faculty of Mathematics, Informatics and Mechanics, University of Warsaw, Banacha 2, Warsaw 02-097, Poland; ‡ Data Science Institute, Hasselt University, Hasselt 3500, Belgium; ¶ Department of Applied Biomedical Science, Faculty of Health Sciences, University of Malta, Msida, MSD 2080, Malta; § Institute for Materials Research (IMO), Hasselt University, Agoralaan, Diepenbeek 3590, Belgium; ∥ IMEC, Associated lab IMOMEC, Wetenschapspark 1, Diepenbeek,3590, Belgium; ⊥ Energyville, Thorpark, Genk 3600, Belgium

## Abstract

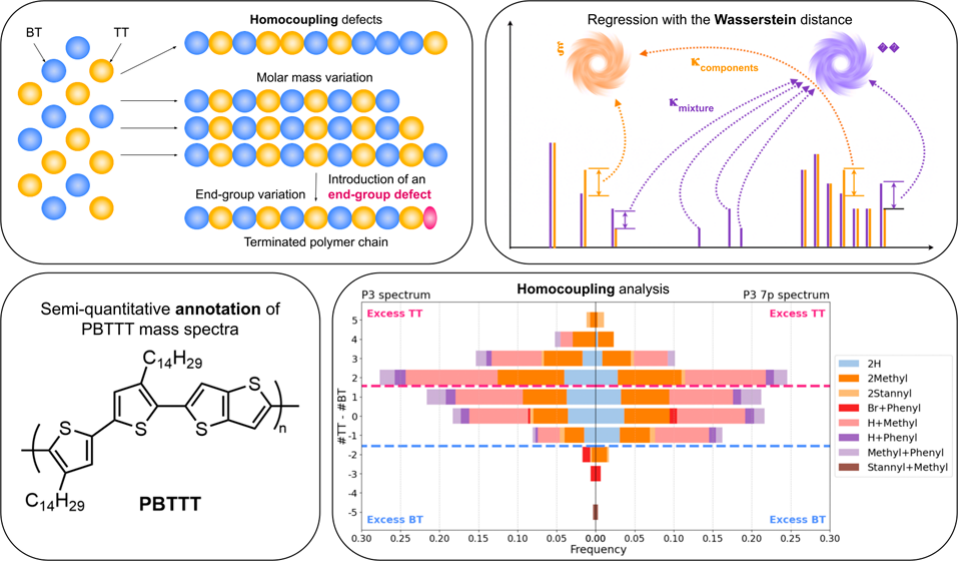

Conjugated organic polymers have substantial potential for multiple applications but their properties are strongly influenced by structural defects such as homocoupling of monomer units and unexpected end-groups. Detecting and/or quantifying these defects requires complex experimental techniques, which hinder the optimization of synthesis protocols and fundamental studies on the influence of structural defects. Mass spectrometry offers a simple way to detect these defects but a manual analysis of many complex spectra is tedious and provides only approximate results. In this work, we develop a computational methodology for analyzing complex mass spectra of organic copolymers. Our method annotates spectra similarly to a human expert and provides quantitative information about the proportions of signal assigned to each ion. Our method is based on the open-source Masserstein algorithm, which we modify to handle large libraries of reference spectra required for annotating complex mass spectra of polymers. We develop a statistical methodology to analyze the quantitative annotations and compare the statistical distributions of structural defects in polymer chains between samples. We apply this methodology to analyze commercial and lab-made samples of a benchmark polymer and show that the samples differ both in the amount and in the types of structural defects.

## Introduction

Conjugated polymers—organic macromolecules characterized by alternating double and single bonds—have received substantial interest over the last decades due to their smooth adjustability in terms of light-harvesting, charge transfer, charge transport and/or emissive characteristics, and their potential for large-scale solution processing at ambient temperatures.^1−3^ Their interesting electronic properties arise from the formation of delocalized π-electrons due to conjugation between the polymer’s repeat units. This allows applications in organic photovoltaics,^4^ organic field-effect transistors,^5^ organic electrochemical transistors,^5^ organic photodetectors,^6^ and organic light-emitting diodes.^7^ Despite these advantages, conjugated polymers have not been commercialized on a mass scale yet, in part due to a number of drawbacks inherent to polymer materials such as batch-to-batch variations, product purity, and structural composition.^8−10^

State-of-the-art push–pull conjugated polymers are mainly synthesized via Stille cross-coupling polymerization of stannylated and brominated (hetero)aromatic monomers.^11−13^ In theory, this should yield perfectly alternating copolymers. In practice, however, the resulting polymer chains often contain structural defects. First, homocoupling of the monomers is observed, which can arise through an oxidative, reductive, or disproportionative pathway, and causes deviations from the desired perfectly alternating structure.^9,14^ Second, the organometallic or halide functionalities present in the monomers are often not incorporated as the terminal groups of the final polymer chains. Instead, side reactions such as transalkylation,^15^ ligand exchange^16,17^ or dehalogenation^18^ can terminate the growing chains, resulting in different end-groups.^9^ Both kinds of structural defects, which often seem system-specific, can result in a shift in the absorption profile and impact crystallinity, blend morphology, and charge transport of the polymer sample.^19−21^

For some polymer systems, the characterization of homocoupling and/or end-groups can be achieved using ^1^H NMR spectroscopy^22−25^ but its broad applicability is hampered by aggregation or limited solubility of conjugated polymers, hence the results are influenced by limited sensitivity and signal broadening. High-resolution scanning tunneling microscopy (STM) imaging does allow for pinpointing and quantifying homocoupling defects but analyzing end-groups with this technique is more difficult due to their relatively small structural alteration to the polymer chain. Neither of these techniques is suitable for a rapid screening of samples of diverse polymer systems to quantify structural defects and fine-tune synthesis protocols.

In 1999, McCullough and his team pioneered the use of matrix-assisted laser desorption/ionization - time-of-flight (MALDI-ToF) mass spectrometry (MS) to softly ionize poly(3-hexylthiophene), allowing for characterization of monomer counts and end-group compositions of individual chains.^26^ MALDI-ToF MS allows for detecting homocoupling in push–pull conjugated polymer chains where the difference between the numbers of each monomer is greater than one. Homocoupling and/or end-groups have been analyzed in several polymer systems using MALDI-ToF MS, although reports remain sparse.^26−31^

Compared to common synthetic polymers, conjugated polymers are relatively short, usually not exceeding a few tens of repeat units and could therefore be considered more of an oligomeric nature. In MALDI-ToF MS, this view is amplified, as shorter chains present in the sample are preferentially detected and longer chains are masked, which could therefore question how representative MALDI-ToF MS structural analysis is.^32^ It was shown for poly[2,5-bis(3-tetradecylthiophen-2-yl)thieno[3,2-*b*]thiophene] (PBTTT), that upon removal of the shorter chains via preparative gel permeation chromatography, defect representation in MALDI-ToF MS was similar both for short and longer chains, thus supporting the relevance of structural analysis on these shorter chains.

One of the major issues in applying MALDI-ToF MS to the analysis of polymer samples on a larger scale is the complexity of the spectra of conjugated polymers, which makes manual annotation difficult, time-consuming and error-prone. Furthermore, as manual annotation is laborsome, already annotated signals will rarely be revisited, which can result in an incomplete or inconsistent annotation. Overcoming this problem could potentially increase the applicability of MALDI-ToF MS for rapid screening of multiple samples, making it easier to adjust and fine-tune new experimental protocols to synthesize polymers with higher quality.

Computational solutions can greatly reduce the manual workload and process more complex spectra. One of such methods, the *regression of spectra* (a type of deconvolution), approximates the analyzed spectrum of a sample as a linear combination of reference spectra of individual compounds in a way that the coefficients of the combination are equal to the proportions of the compounds.^33,34^ PyMacroMS^35^ and COCONUT^36^ are examples of regression models developed for polymer samples, which are based on the popular ordinary least-squares approach. The Masserstein package^34^ is an example of a general-purpose tool based on a conceptually different approach, i.e. minimizing the Wasserstein distance between the spectrum and the combination. While the ordinary least-squares approach compares the intensities of peaks with the same mass to charge (*m*/*z*) values, the Wasserstein distance measures the total shifts in *m*/*z* values between corresponding signals,^37^ thus better handling measurement errors and different resolutions of the compared spectra.^38^

Regardless of the particular computational approach, annotation of polymer spectra requires a complex pipeline that involves preprocessing the spectra and postprocessing the regression results. Even a perfect regression model will provide inaccurate results if other steps of the pipeline are not optimized, or if the parameters of the used programs are set incorrectly. Furthermore, a thorough annotation requires the use of large compound libraries. This raises the risk of false annotations because combining more spectra allows the model to better fit the data, even if the added spectra are not chemically relevant. Developing a regression model that can handle large libraries would decrease the risk of false positives and increase the applicability of this approach.

In this work, we develop and optimize a computational pipeline for analyzing MALDI-ToF MS spectra of alternating conjugated polymers to statistically characterize their structural defects without the need for detailed knowledge of the polymer’s structure. Our method does not require chromatographic separation of the sample nor tandem MS fragmentation of polymer chains. We compare the performance of Masserstein and PyMacroMS^35^ on commercial and lab-made samples of poly[2,5-bis(3-tetradecylthiophen-2-yl)thieno[3,2-*b*]thiophene] (PBTTT), a benchmark semicrystalline polymer which is known to show homocoupling defects.^39,40^ We develop a statistical methodology to use the automated annotation for analyzing the prevalence of homocoupling and the end-group composition of conjugated polymer chains. We also develop an extension for the Masserstein package to handle large libraries of reference spectra. We give a detailed description of how to properly tune the software parameters and preprocess the spectra in order to obtain optimal results. Our pipeline, in the form of Jupyter notebooks, is available in the Tutorials section at https://github.com/ciach/masserstein.

## Materials and Methods

### Data Set

A sample of PBTTT (C14), denoted P1, was synthesized according to an adapted literature procedure (Suppl. Figure S1), combining a dibrominated bithiophene (BT) and a distannylated thieno[3,2-*b*]thiophene (TT).^39,41^ Additionally, two commercial samples (P2, P3) were purchased from Sigma and Lumtec, respectively. The molar mass characteristics can be found in Supplementary Table S1. MALDI–ToF mass spectra were recorded on a Bruker Daltonics UltrafleXtreme MALDI/TOF-TOF system. Ten μL of the matrix solution consisting of 20 mg/mL *trans*-2-[3-(4-*tert*-butylphenyl)-2-methyl-2-propenylidene]malononitrile (DTCB) in chlorobenzene was mixed with 3.5 μL analyte solution of 1 mg/mL in chlorobenzene. Then, 1 μL of said mixed solution was spotted onto an MTP Anchorchip 600/384 MALDI plate. Mass spectra of the three samples, denoted P1–P3, were recorded at a laser fluence setting of 15%. Additionally, one spectrum, denoted P3_7p_, was recorded from the same sample as P3 but at a laser fluence setting of 7%.

### Extending the Masserstein Algorithm to Handle Large Reference Libraries

Let μ be the experimentally measured spectrum of a mixture of polymer compounds, referred to as the mixture spectrum. Let ν_1_, ..., ν_*k*_ be a library of reference spectra (full isotopic envelopes) of individual polymers with given end-groups, so that each ν_*i*_ spectrum is associated with a unique molecular formula (with the spectra either predicted theoretically or acquired experimentally). Assume that all the spectra are normalized by their total intensity.

Following ideas outlined in previous works,^42^ we construct a model spectrum ν_*p*_ = *p*_1_ν_1_ + *p*_2_ν_2_ + ··· + *p*_*k*_ν_*k*_, where *p*_*i*_ is the proportion of ν_*i*_ in μ to be estimated. To account for a signal present in μ but absent in ν_*p*_ (background noise, contaminants, analytes not included in the library, etc.), we construct an ”augmented model spectrum” ν_*p*_ + *p*_0_ω, where ω labels the excess signal in μ and *p*_0_ is its proportion. To account for signal missing in μ but present in ν_*p*_ (signals below the limit of detection, contaminants in experimentally acquired library spectra, etc.), we construct an ”augmented mixture spectrum” (1-*p*_0_^′^)μ + *p*_0_^′^ξ, where ξ is the signal missing in μ (equivalently, contaminating signal in ν_*p*_), and *p*_0_^′^ is the proportion of such signal.

Labeling signal as contaminating or missing is controlled by two user-specified denoising penalties κ_mixture_ and κ_components_, the former being equal to the cost of removing unit signal from μ, and the latter from ν_*p*_. Both parameters correspond to the expected maximum distance, expressed in *m*/*z* units, between the matching experimental and theoretical signals.^34,42^ However, as we will show, an optimal value of κ_components_ is slightly higher than κ_mixture_, especially in the case of theoretically predicted reference spectra. The reason is that surplus signals in experimental spectra (i.e., any signals not included in the reference library, e.g. background noise, additional polymer chains) often add up to a higher amount of intensity compared to missing signals (i.e., fragments of isotopic envelopes of ions which are present in both the experimental spectrum and the reference library, e.g. signals below the limit of detection). Thus, unless the experimentally measured spectrum is free from any background noise and contaminants, and the reference library is fully comprehensive, the amount of signal that needs to be removed from the experimental spectrum is likely to be higher than the amount of signal that needs to be added to it (if any) in order to obtain agreement with the model spectrum. Setting κ_components_ higher than κ_mixture_ also reflects the prior belief that while the model can focus on a selected subset of the experimentally measured data, it should not be allowed to assume the presence of any additional signals in the data unless this assumption is necessary to obtain agreement with theory and well-supported by other observations (e.g., when a signal is part of an otherwise present isotopic envelope but is on the level of background noise).

To accommodate for large libraries, we modify the model above as follows. Assuming that there is a proportion *p*_*i*_ of compound *i* in the mixture spectrum inflicts an additional cost equal to *p*_*i*_*c*_*i*_, where the annotation penalty *c*_*i*_ is a user-defined parameter. This way, *c*_*i*_ reflects the prior knowledge about how unlikely the *i*-th compound is to be found in a spectrum, and the default value of *c*_*i*_ = 0 can be interpreted as “no prior argument against the occurrence of this compound”. This prior knowledge makes the model robust against spurious annotations. Notably, this approach still allows the model to annotate the spectra with less likely compounds, but only if the experimental evidence for their presence is sufficiently robust. Therefore, it is more flexible than simply discarding the less likely compounds from the library. The total penalty for annotation is equal to *c*_*p*_ = *p*_1_*c*_1_ + ··· + *p*_*k*_*c*_*k*_.

Now, we can estimate the vector of proportions *p*, the proportion of excess signal *p*_0_, the proportion of additional signal *p*_0_^′^, and the spectra of contaminating and missing signals ω and ξ by fitting the ”augmented model spectrum” to the ”augmented mixture spectrum”. This can be accomplished by minimizing the Wasserstein distance between the two augmented spectra plus the additional penalty for large libraries:
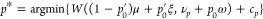
1The values of the annotation penalties *c*_*i*_ can be estimated in a straightforward manner: for two competing spectra, say ν_1_ and ν_2_, setting *c*_1_ – *c*_2_ > *W*(ν_1_, ν_2_) guarantees that the spectrum is annotated with ν_2_; setting *c*_2_ – *c*_1_ > *W*(ν_1_, ν_2_) guarantees that the spectrum is annotated with ν_1_; and setting |*c*_1_ – *c*_2_| < *W*(ν_1_, ν_2_) allows the model to have a more flexible choice between ν_1_ and ν_2_. Therefore, the range of reasonable penalties for such competing compounds lies between zero and *W*(ν_1_, ν_2_).

In the Supporting Information, we provide a more rigorous description of the optimization problem (1). We implemented an algorithm solving it with the Simplex method and added it to the open-source Python 3 package Masserstein available at https://github.com/mciach/Masserstein/.

### Semiquantitative Annotation of Spectra

**Figure 1:**
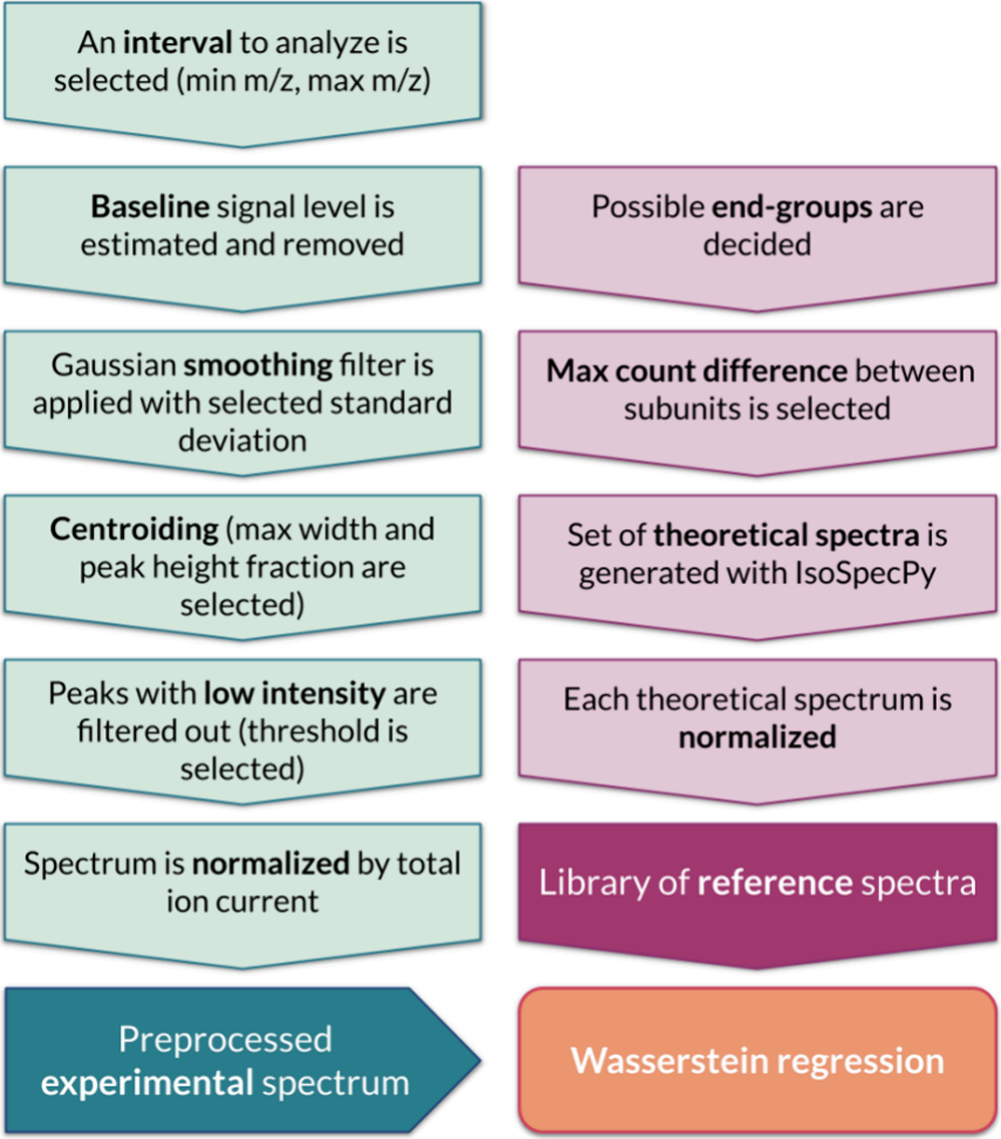
Workflow of our pipeline for preprocessing the experimental spectra, generating theoretical spectra, and performing a Wasserstein regression of spectra with Masserstein.

A fully quantitative mass spectrometric analysis is difficult or impossible for many polymer systems due to multiple factors, such as differences in ionization tendencies of different molecules.^9,43^ However, the proportion of signal attributed to a given polymer species can be used as a rough, semiquantitative measurement of its concentration. The main steps of the pipeline for annotation of spectra and estimation of the polymer proportions are shown schematically on the workflow in Figure 1. The same workflow was used for the Wasserstein regression with Masserstein and the Ordinary Least Squares regression with pyOpenMS.^44^ The steps of the workflow are described in detail in the following paragraphs.

### Preprocessing Experimental Spectra

We restricted the spectra to the 3000–4500 *m*/*z* mass range to exclude regions with low signal-to-noise ratio and excessive baseline. Next, we removed the baseline signal by visually inspecting the spectra for the noise level and subtracting it from the spectra. After that, a Gaussian filter (σ = 0.1) was applied to smooth out the spectra and decrease the electronic noise. Following the smoothing, the spectra were centroided using the centroiding function from the Masserstein package.^34^ The centroiding procedure from Masserstein integrates the peaks within regions delineated by a user-specified fraction of the apex intensity (set here as 0.5, corresponding to integration within the full width at half-maximum, fwhm), and discards peaks as spurious if their width is excessive (fwhm threshold 0.75 Da). In principle, this step is not necessary, because Masserstein by design allows for using spectra in profile mode and even mixing profile and centroided spectra. However, centroiding increases the computational efficiency and decreases the risk of false positive results.^34^ After centroiding, in each spectrum, peaks with intensity below a fraction of 0.001 of the highest peak were discarded. Finally, all the spectra were normalized by their total intensity.

### Generating Reference Spectra

Since both Masserstein and pyMacroMS support generating libraries of reference spectra, we generated a library for each package using its built-in functions. For Masserstein, we generated a library of reference spectra consisting of the full theoretical isotopic envelopes of all polymer species (charge = 1, no adduct ions) with masses within 3000–4500 Da with a maximum count difference between monomers equal to 5 (359 spectra). We considered the following end-groups: bromine (default BT end-group), trimethylstannyl (default TT end-group), methyl, hydrogen, and phenyl (side reaction end-groups). We excluded polymers with inconsistent end-group and monomer composition, such as 10TT+2Br. We discarded isotopic envelopes encompassing less than 0.1% of the total experimental intensity by setting the MDC parameter of Masserstein to 0.001 in order to speed up the computations. Since the estimated proportions of such ions are bounded by 0.1%, they have a minor contribution to the overall statistical results, and we decided to ignore them in our study design.

For pyMacroMS, the molecule library was simulated using the Polymer class for the custom end-groups database and custom monomer database for the PBTTT polymers (charge = 1, no adduct ions), created similarly to the analysis performed by De Bruycker et al.^35^ Both pyMacroMS and Masserstein use the IsoSpec algorithm^45^ to calculate isotopic distributions.

### Tuning Masserstein Parameters

To find appropriate values of parameters needed to run Masserstein, the reference library was fitted to spectra P1 and P2 for all combinations of κ_mixture_ and κ_components_ values from a range of 0.1, 0.2, ..., 0.9. The fitted model was assessed visually in the *m*/*z* regions 3335–3374, 4244–4273 in the P1 spectrum and 3190–3240, 3333–3377, 3410–3442 in the P2 spectrum. Parameter values κ_mixture_ = 0.6 and κ_components_ = 0.7 provided a good visual agreement between the fitted model and the experimental spectra. Spectra P3 and P3_7p_ were not used during fitting but were used to evaluate the method later on. After estimating polymer proportions, polymer species with estimated proportions below a threshold of 0.2% of the total experimental signal were discarded. The purpose of this step was to decrease the number of false annotations by removing polymers with an estimated intensity close to the level of background noise.

Without added *c* penalties for annotation, Masserstein struggled with discerning between polymer species with formulas *m*BT+*n*TT+H+Methyl and *m*BT+(*n* – 1)TT+2Phenyl. This was because of the high similarity in the masses of such compounds, with the difference in exact monoisotopic masses equal on average to only 0.05 Da, and the Wasserstein distance (interpreted as a combined difference in exact masses of all peaks) equal on average to 0.1905 Da for *n*, *m* between 1 and 8 (see Suppl. Figure S2 for an example pair of isotopic envelopes with *n* = *m* = 5, Wasserstein distance 0.191 Da). Such ions are difficult to unambiguously annotate using many commonly used instruments. For example, for m = n = 5, the theoretically predicted monoisotopic masses of these compounds are 3211.979 and 3212.066. Any visible separation of such masses would require a resolving power over 37 000 at 3.2 kDa. An unambiguous assignment and quantification of intensity would require it to be significantly higher - for example, obtaining a valley separating the two peaks of at least half the peak height would require a resolving power of 53 000 at 3.2 kDa.

With the added penalty *c* = 0.1905 for all polymers with two phenyl end-groups, a clear pattern was observed where *m*BT+*n*TT+H+Methyl compounds were neighboring with *m*BT+*n*TT+2H and *m*BT+*n*TT+2Methyl compounds, and, as would be expected, *m*BT+*n*TT+H+Methyl ones had twice as high intensity than the other two. Without the added penalty, no such pattern was observed neither for *m*BT+*n*TT+H+Methyl nor for *m*BT+(*n* – 1)TT+2Phenyl compounds (Suppl. Figure S2). Therefore, for annotations and downstream analyses, we set a penalty of 0.1905 for polymers with two phenyl end-groups, keeping other penalties equal to zero. Note that different experimental conditions may require a different value of this penalty.

### Tuning pyMacroMS Parameters

A grid search was performed for the parameters minRelAbundance ranging from 0.005 to 0.05, and ppmDev from 5 to 20. The ppmDev parameter is the maximum allowed deviation between matching experimental and theoretical peaks (i.e., similar to the κ parameters of Masserstein but expressed in ppm rather than *m*/*z* units), and the minRelAbundance parameter is the threshold below which experimental signal is considered as noise. The minRelAbundance parameter is also used when generating reference spectra so that only the isotopes with a relative abundance larger than minRelAbundance are included.

The annotations seemed relatively stable for a broad range of parameters, with no single set of values that would give a clearly better performance than others (Suppl. Figure S8). We selected ppmDev = 16 since it seemed to give stable enough results independent of the selected minRelAbundance value, minRelAbundance = 0.03, and resolution = 80000.

### Evaluating Performance of Regression Approaches

We compared the automatic annotation with an annotation generated manually by an expert using the Jaccard score, defined as the size of the intersection divided by the size of the union of the sets of polymer species annotated by the expert and by the software. The Jaccard score is equal to 1 when both annotations are identical, and equal to 0 when they are fully disjoint. We also calculated the sensitivity of the annotation, defined here as the number of polymer species annotated by both the software and the expert divided by the number of polymer species annotated by the expert. The sensitivity is equal to its maximum value of 1 when the software detects all the polymer species annotated by the expert (possibly including some additional polymers missed by the expert), and is equal to its minimum value of 0 when the software fails to detect any annotations of the expert.

### Homocoupling Analysis

Estimating polymer proportions with Masserstein produces results in the form of (#TT_*i*_, #BT_*i*_, *E*_*i*_, *p*_*i*_), where #TT_*i*_ is the number of TT subunits in the *i*-th polymer, #BT_*i*_ is the number of BT subunits, *E*_*i*_ is the set of end-groups (we do not differentiate between the two ends of a polymer chain), and *p*_*i*_ is the proportion of the overall signal in the spectrum matched to the *i*-th polymer. In order to remove the influence of unassigned signals from *p*_*i*_, we normalize the proportions as *p*_*i*_^*norm*^=*p*_*i*_/∑_*j*_*p*_*j*_, so that *p*_*i*_^*norm*^ is the proportion of the *i*-th polymer among the annotated polymer species rather than among all ions in the spectrum. To calculate the total proportion of polymers with *m* TT subunits and *n* BT subunits, denoted as *P*(#TT = *m*, #BT = *n*), we sum the corresponding normalized proportions over all end-groups:
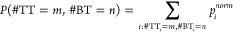
2To quantify the total proportion of polymers with *m* TT subunits, *P*(#TT = *m*), the values of *P*(#TT = *m*, #BT = *n*) are summed over all values of *n*. Note that #TT can be interpreted as the number of TT subunits in a randomly selected polymer chain. Similarly, to quantify the total proportion of polymers with *n* BT subunits, *P*(#TT = *m*, #BT = *n*) are summed over all values of *m*. Finally, to quantify the proportion of polymer species with a given difference between the numbers of subunits, *P*(Δ = *d*), the proportions of all the corresponding polymers are summed:
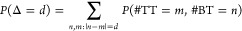
3For a perfectly alternating copolymer, the proportion of species with Δ = 1 (i.e., an excess of 1 TT subunit, corresponding to a TT subunit at both ends of the polymer) is expected to be equal to . Symmetrically, , corresponding to a BT subunit at both ends, and , corresponding to different subunits at both ends. However, if the analyzed sample contains homocoupled polymers, we expect to see shifts in the distribution of the Δ statistic, in particular toward values above 1 and/or below −1. This allows us to statistically analyze homocoupling defects without detailed knowledge of the structure of each polymer species observed in the spectrum.

## Results and Discussion

### Separating Complex Clusters of PBTTT Molecular Species by Fitting Isotopic Envelopes

**Figure 2:**
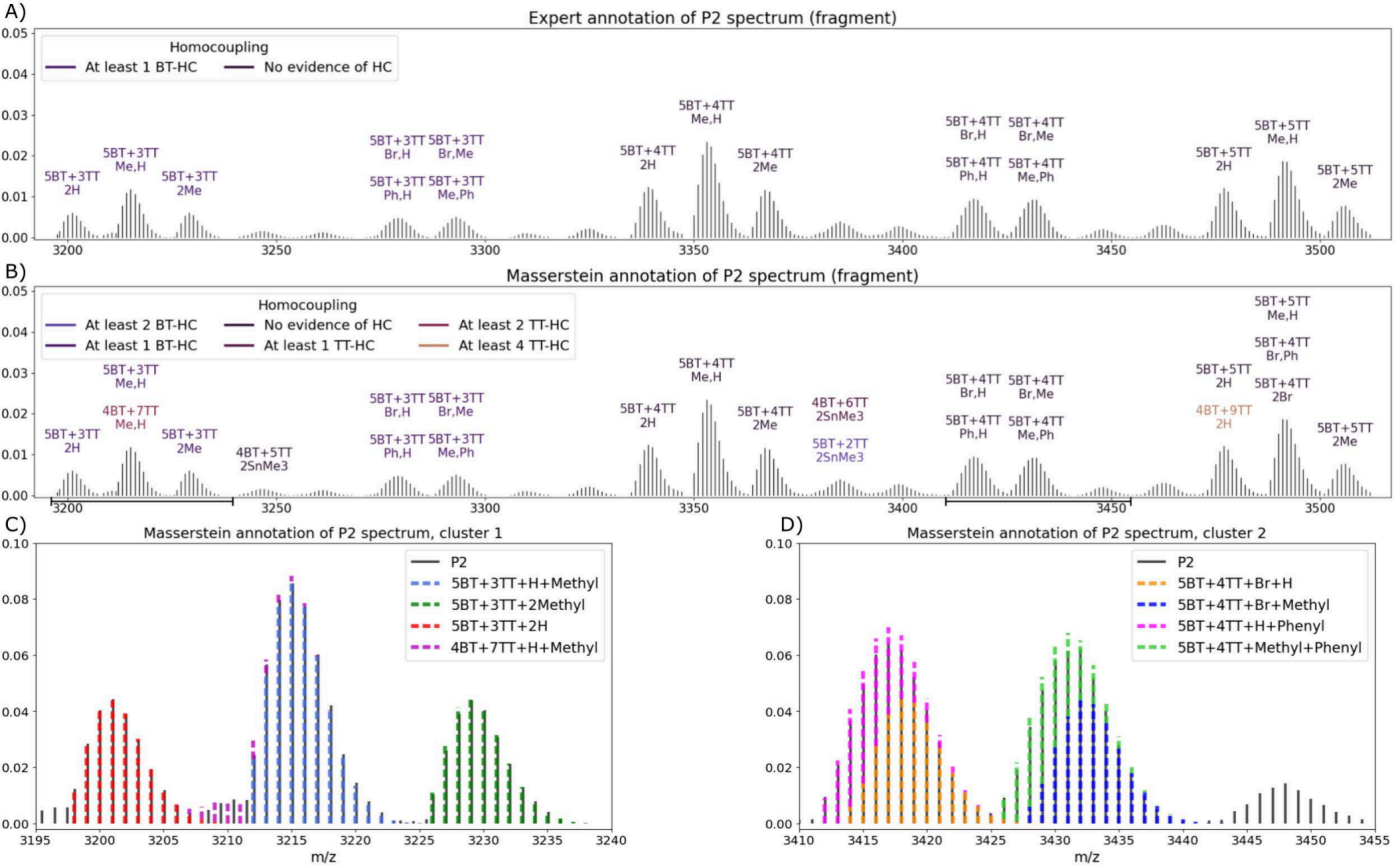
Annotation of a fragment (3300–3650 *m*/*z*) of PBTTT polymer mass spectrum P2 done by a human expert (A) and Masserstein (B). Examples of Masserstein annotations of complex clusters of overlapping isotopic envelopes are shown in (C) and (D). Cluster 2 in (D) was annotated in the same way by both Masserstein and the expert, while in (C) the software detected additional polymer chains 4BT+7TT+H+Methyl. Horizontal dimension bars in (B) highlight the locations of the zoomed-in clusters.

The Jaccard score of Masserstein annotation ranged from 46% for P1 to 65% for P3 (Figure 2, Suppl. Figures S3–S7). Most of the molecular species annotated by the expert but missed by Masserstein were below the proportion threshold of 0.002. These species constituted a minor proportion of the total signal, and consequently, between 82% and 94% of the total signal in the spectra was annotated correctly. On the other hand, the Jaccard score for annotations obtained with pyMacroMS was considerably lower, ranging from 0% to 44% (Suppl. Figure S7). While other combinations of minRelAbundance and ppmDev parameters improved the score for individual spectra, no combination improved the overall score in all spectra (Suppl. Figure S8). Consequently, for subsequent statistical analyses of structural defects, we use the results obtained with Masserstein.

Due to the number of possible end-groups, the isotopic envelopes of different molecular species of PBTTT can overlap to the extent that they appear as single visually identifiable peaks, making it challenging to accurately annotate the spectra manually using only the masses of polymers. In contrast, fitting whole isotopic envelopes of all components with Masserstein provided a detailed annotation of complex clusters and, additionally, a quantification of the proportions of individual components. The fitted models agreed visually with the experimental spectra. (Figure 2). The fact that a human expert tends to assign one annotation to a single visually identifiable peak, while Masserstein provided detailed annotations, decreased the overall Jaccard score of the annotation. The sensitivity of annotation was noticeably higher, ranging from 70% for P1 to 75% for P3.

To double-check these conclusions, an expert has evaluated randomly selected annotations provided by Masserstein. Out of 42 inspected annotations, 33 were confirmed, 7 were ambiguous, and one was deemed incorrect (5BT+2TT+Br+Phenyl). The ambiguous ions were mostly polymers containing stannyl end groups. As we discuss later on in the section about end-group analysis, these ions are generally difficult to annotate, but an additional analysis suggested that these annotations are correct.

Both programs returned some number of annotations that seem unlikely from a chemical point of view, such as 2BT+14TT+Br+Methyl returned by pyMacroMS or 4BT+9TT+2Br returned by Masserstein. The former formula would indicate a very high degree of homocoupling, which in turn would suggest a block structure rather than an alternating one, which is not an expected product of the Stille condensation reaction. The latter formula is unlikely because bromine is the default end-group for BT subunits, effectively also suggesting a block copolymer. This signifies that while the automatic assignment tools can greatly simplify the annotation of spectra and discover more molecular species in complex clusters, their results still require manual verification for chemical plausibility.

### A Statistical Analysis of Homocoupling Patterns and Polymer End-Group Composition

In this subsection, we use polymer proportions estimated with Masserstein for a statistical analysis of the structural characteristics of polymer samples.

#### Differences in Complexity of Polymer Samples

We detected between 57 and 90 different molecular species in the PBTTT spectra (Supplementary Table S2), corresponding to between 83% and 91% of the total signal. Spectrum P2 had both the highest number of molecular species detected and the highest amount of signal that could not be matched by any polymer species from the library, indicating a highly complex sample. On the contrary, spectrum P1 had both the highest amount of signal matched to the library and the lowest amount of detected molecular species, indicating a sample with a more homogeneous composition. Spectra P3 and P3_7p_, obtained for the same sample but with different laser luminosities, had nearly identical annotations, indicating a relatively low variation of the measurement and estimation procedures.

#### Differences in Prevalences and Types of Homocoupling Defects

The statistical methodology introduced in the Methods section allows us to rigorously compare structural defects between samples. As expected, the numbers of BT and TT units in polymer chains were linearly correlated in all samples, but some structural defects were present as well (Suppl. Figure S9). The proportion of homocoupled polymer molecules (|Δ| > 1) was lowest for P1 and highest for P3  for P1, 28% for P2, 52% for P3 and 41% for P3_7p_).

**Figure 3:**
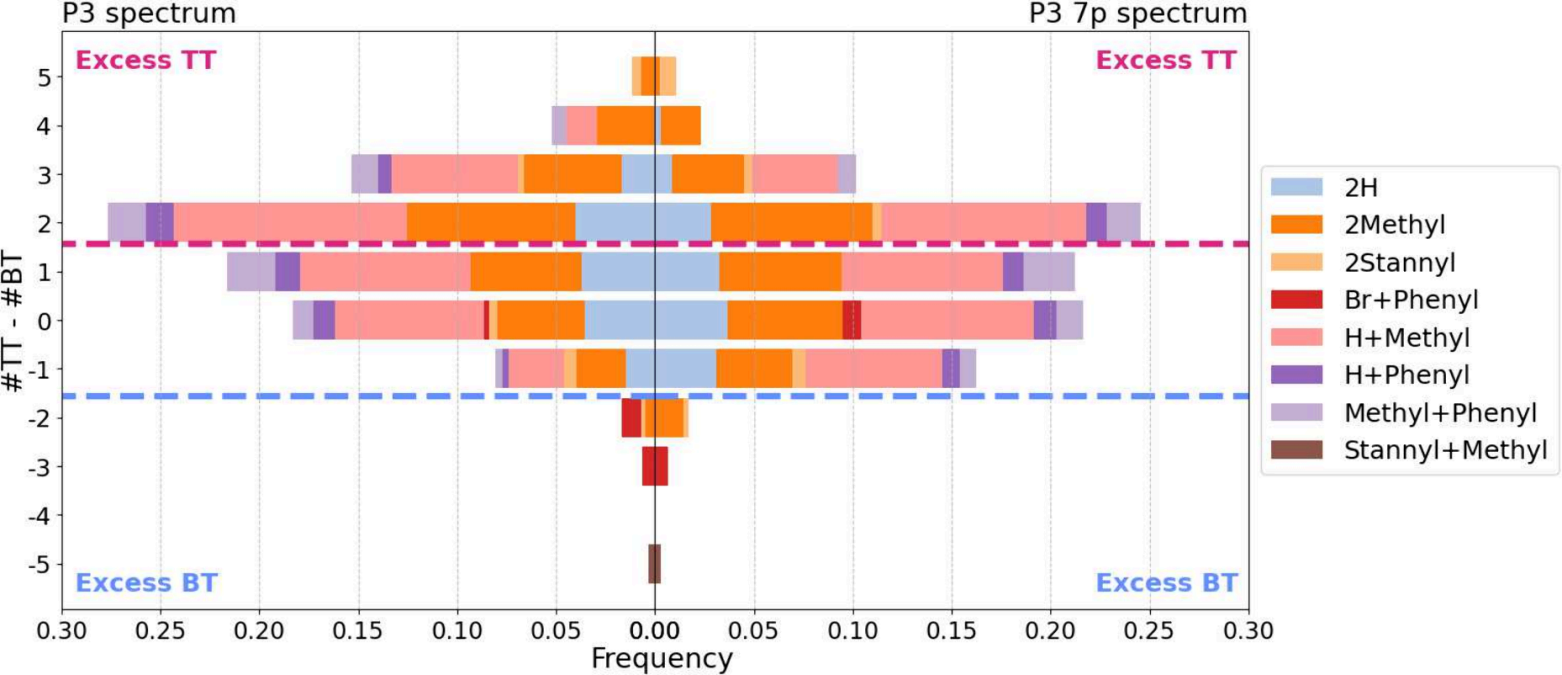
Distribution of the difference of subunit counts, Δ = #TT – #BT, in all polymer species annotated with Masserstein in spectra P3 (left) and P3_7p_ (right). The bars are colored according to the end-group composition of polymers. Values of Δ above zero (top half) indicate an excess of TT subunits, while values of Δ below zero indicate an excess of BT subunits; values greater than 1 or lower than −1 indicate homocoupling. The distribution of Δ for P3_7p_, obtained on the same sample but with a lower laser luminosity, is slightly shifted to lower values compared to P3. At the same time, the end-group compositions are similar for both spectra, indicating little to no effect of luminosity.

In samples P3 and P3_7p_, a shift of the Δ*d*istribution toward values above 1 suggests that homocoupling was mostly caused by the excess of TT units (Figure 3, Suppl. Figure S9). This is further corroborated by the distributions of the counts of TT and BT units (Suppl. Figure S10). However, these results should be interpreted with caution, as MALDI-ToF MS may overestimate the amount of TT homocoupling.^40^. In the P2 spectrum, homocoupling of BT subunits seemed more common, visible as a left-skew of the distribution of Δ. The distributions of subunit counts also show that #TT has a considerable variance, while #BT was relatively stable in these samples (Suppl. Figure S10).

Sample P1 had a profoundly different shape of the histogram of the Δ*s*tatistic, with prominent peaks for polymers without evidence for homocoupling, especially for BT-capped chains ;  and . However, the distribution also showed an unexpected, separate subpopulation of polymer chains with an excess of three to five TT subunits, constituting 10% of signal (Suppl. Figures S9, S10). We suspect that this subpopulation may be an artifact caused by spurious annotations by the software or an incomplete library of reference spectra. This finding points to the importance of inspecting histograms of distributions in addition to simple statistics to detect anomalous results.

#### Analyzing End-Group Composition of Polymers

The most common end-groups of polymer species in P1 were two hydrogen groups (25% of the annotated polymer signal), two bromine groups (17%), and hydrogen–bromine groups (30%); a noticeable amount of polymers also contained one or two methyl end-groups (21% of signal; Suppl. Figures S11, S12). In the remaining samples, the polymer species capped with one hydrogen and one methyl end-group were the most common (32–39%), followed by two hydrogens (14–19%) and two methyls (14–31%); the two bromine end-groups were almost absent (<1%). Spectra P3 and P3_7p_ had a nearly identical end-group composition, indicating that laser luminosity does not significantly influence which end-groups are more prevalent in the spectrum (Figure 3, Suppl. Figure S11). This also supports the conclusion that differences observed between the samples are due to variations in synthesis methods rather than measurement errors. We also detected polymer species containing a phenyl end-group in all the spectra (6–15%).

In P1, the aforementioned subpopulation of polymer chains with an excess of three to five TT subunits had bromine end-groups (Suppl. Figure S12). Since bromine is the default end-group for the BT subunit (Suppl. Figure S1), this result supports the hypothesis that this subpopulation may be an artifact of the annotation. However, inspecting the fitted model for these polymers shows that they agree very well with the data (Suppl. Figure S13), and we found no other polymer species in our data set that had similar masses to those chains. Therefore, if this result is indeed a mistake of the software, it is not an obvious one.

In both P3 and P3_7p_, the software detected a small amount of BT-homocoupled polymer chains with a stannyl end-group (Figure 3). This result is seemingly counterintuitive, as stannyl end-groups are associated with TT subunits (Suppl. Figure S1), and might suggest a possible mistake of the software, especially since the proportions of those chains are close to the noise level. However, a closer examination of the distribution of the Δ statistic for all polymers with one or two stannyl end-groups, including the discarded species below the proportion threshold of 0.002, showed that heavily BT-homocoupled chains tend to have only one such end-group (Suppl. Figure S14), while higher values of Δ (up until Δ = 0) were associated with higher proportions of chains with two, rather than one, stannyl end-groups, as would be expected. This association suggests that these may be *bona fide* annotations and thus a low proportion of heavily BT-homocoupled chains with a stannyl end-group may be present in the sample. However, we would like to stress that in general, stannyl end-capped species are difficult to accurately assign due to their broad isotopic envelopes. A crosscheck with other characterization techniques such as NMR might be relevant if these species are the specific focus of the MALDI-ToF MS analysis. Nevertheless, this statistical association between homocoupling defects and end-group composition, if confirmed, would reveal an additional layer of complexity of polymer samples. Capturing the full picture of this association would require more elaborate mathematical modeling that is beyond the scope of the present work.

### Optimizing the Pipeline for Regression of Spectra

As shown in the workflow in Figure 1, estimating polymer proportions with regression of spectra requires a series of preprocessing steps that form a complex pipeline. Since errors can propagate through the pipeline, a proper execution of each step, including parameter tuning, is necessary to obtain proper results. To facilitate the use of regression of spectra in future analyses, we finish this article by describing our results in optimizing the pipeline.

#### Masserstein Annotation Is Robust for a Wide Range of Parameters

Our results were obtained for κ_mixture_ and κ_components_ selected manually by inspecting different values and selecting the ones that give a visual agreement between the model and the experimental spectrum in manually selected clusters of overlapping isotopic envelopes. This way, the parameters can be selected without looking at the expert annotation, avoiding bias and limiting the associated manual workload. In this section, we run additional experiments to ensure that this procedure gives correct parameter values and investigate what level of precision is required when tuning them and whether a single set of parameters can be used for all the spectra.

To examine the influence of κ_mixture_ and κ_components_ on the results, we calculated the Jaccard scores for parameter values ranging from 0.1 to 0.9. The results showed that the manually selected parameter values provided accurate results for all the samples. We observed a “basin of optimality” of the parameter values, where the Jaccard score remained stable for a relatively broad range of parameters (Suppl. Figure S15). However, a gross misspecification of the parameters resulted in highly incorrect results. This shows that a manual tuning of the Masserstein parameters is necessary before using the software, but does not require excessive precision and can be done by a simple visual inspection of the fitted model.

The optimal values of the κ parameters may depend on multiple factors, such as the peak width (resolving power), the number of peaks in the isotopic envelope and its size, the molecule’s charge, signal-to-noise ratio, and ion statistics. The default parameter values in Masserstein are a reasonable starting point, but it is always highly recommended to inspect fitted models for at least a handful of parameter values. Alternatively, the parameters can be tuned by optimizing the Jaccard score on a handful of manually annotated spectra of the same system measured under the same conditions. For most applications in mass spectrometry, the reasonable range of κ values to inspect is between zero (perfect agreement between theoretical and experimental spectra) and one (up to 1 Da difference between matching theoretical and experimental peaks), typically keeping κ_components_ moderately higher than κ_mixture_.

#### Low-Intensity Compounds Are Difficult to Annotate, Especially in the Presence of Overlapping Isotopic Envelopes

One of the reasons why the automatic annotation struggled with low-intensity compounds was that we discarded compounds whose proportions were below the minimum threshold. While disabling the threshold allowed the sensitivity to reach almost 100% for P2, it also produced numerous seemingly spurious annotations which overall resulted in a decrease, rather than an increase, of the Jaccard score. One of the reasons is that regression-based approaches tend to add additional, low-intensity compounds to complex clusters of signals in order to fill the clusters as well as possible. Optimal Jaccard scores were obtained for threshold values between 0.001 and 0.004 in all four spectra (Suppl. Figure S16).

#### Proportion Threshold and Annotation Penalty Complement Each Other

The proportion threshold and the annotation penalty are two ways to limit the number of false positive annotations: the penalty sets a constraint on the number of annotated polymer species during the estimation, while the threshold discards polymer species with insufficient proportions after the estimation. While both these parameters have the same goal, they achieve it in different ways which complement each other. As a consequence, the optimal Jaccard score is typically achieved when both are used jointly. However, good results are obtained for a wide range of parameter values as long as at least one of these filters was used (Suppl. Figure S17). Currently, the recommended way to select the parameter values is to fit a model, inspect it visually, assess the presence of false low-intensity annotations, set the threshold and/or the annotation penalty accordingly, and repeat the procedure to check for improvement. Alternatively, as with the κ parameters, the proportion threshold and annotation penalty can be tuned by optimizing the Jaccard score on a handful of manually annotated spectra. Further research is necessary to optimize the protocols for tuning the penalty and threshold parameters in a more convenient way.

#### A Proper Library Is Crucial for Proper Results, and Its Size Has a Bias-Variance Trade-Off

While large libraries of reference spectra allow for discovering more molecular species, using them is computationally expensive and can lead to false positive annotations. The reference spectrum of a molecule absent from the sample always has some chance of matching a random noise signal, an isobaric interference, or a complex cluster of isotopic envelopes producing a false positive. Nevertheless, selecting too small of a library can lead to false negative results where some analytes are not detected.

These considerations point to a bias-variance trade-off in library-based quantification methods: insufficient libraries systematically underestimate the number of polymer species, while excessive libraries have a higher risk of spurious annotations caused by assignments of analytes to random signals. We tested this trade-off empirically by generating an ”extended” reference library, with a maximum difference of subunit counts up to 10 (570 spectra), and a ”restricted” reference library, with a maximum difference of up to 2 (163 spectra). As expected, the extended library resulted in additional annotations. However, these additional annotations were often added to complex clusters of isotopic envelopes and only marginally improved the visual agreement between the experimental spectrum and the model, while containing rather unlikely, highly homocoupled polymer chains such as 3BT+13TT, artificially inflating the homocoupling measures (Suppl. Figure S18). Conversely, the truncated library resulted in missing some obvious signals and resulted in underestimated homocoupling. The library with a maximum count difference up to 5 resulted in optimal annotations and was therefore selected for this work.

## Conclusions

Understanding the structure of alternating conjugated polymers can be challenging, often relying on assumptions based on the introduced monomers. These assumptions are not always satisfied, as side reactions can complicate the structure of the polymer chains, leading to unexpected species in the final sample. Despite its importance for the quality of the end product, the structural quality of the synthesized polymers is rarely assessed thoroughly, partly due to the complexity and limitations of the required instrumentation and analysis methods.^40,46^

MALDI-ToF MS offers a way to gain insight into the composition of the sample by differentiating polymeric species based on their masses. However, a manual analysis of the often complex spectra of conjugated polymers can be laborious and time-consuming. Our contribution addresses this issue by not only annotating complex clusters of overlapping isotopic envelopes but also providing semiquantitative information about the signal contribution of each species, a step rarely taken with manual assignments. This automation eliminates the tedium of manual analysis, allowing for the discovery of more molecular species and reducing the likelihood of oversimplified assignments. We thoroughly analyzed the potential sources of errors associated with our methodology and developed an optimized protocol to avoid them.

The statistical methodology developed as a part of this work allows for transforming the semiquantitative annotations into robust comparative studies of MALDI-ToF mass spectra, offering insights into the technique’s properties and the chemistry of the polymers. By identifying specific end-groups and homocoupling defects, this approach can be used to gain a deeper understanding of the material’s composition and behavior. This can be particularly powerful to probe the activeness of chains after a prepolymerization step as for instance required in the multistep synthetic protocol for conjugated block copolymers.^47^ Our streamlined approach allows for efficient characterization of polymer samples, making it a valuable tool for researchers seeking to gain insights into polymer structures without the need for complex experimental techniques.

## Supporting Information Available

Full results, supplementary figures, and the derivation of the algorithm for estimation of analyte proportions through the Wasserstein regression with penalty for annotations with large libraries (PDF)

## Notes

The Python package with algorithm implementation and the Jupyter notebooks with our analysis are available at https://github.com/ciach/masserstein.
