# Supplementary material for: An Automated Analysis of Homocoupling Defects Using MALDI-MS and Open-Source Computer Software
Source: J Am Soc Mass Spectrom. 2024 Sep 18;35(10):2366–75. doi: 10.1021/jasms.4c00225 (PMC11450816; doi:10.1021/jasms.4c00225)
Supplement: Supplementary file 1 — js4c00225_si_001.pdf [file js4c00225_si_001.pdf]

Supporting Information

An automated analysis of homocoupling defects using MALDI-MS and open-source computer software

Maria Bochenek,[†] Michał Aleksander Ciach,[†,‡,¶] Sander Smeets,[§,‖,⊥] Omar Beckers,[§,‖,⊥] Jochen Vanderspikken,[§,‖,⊥] Błażej Miasojedow,[†] Barbara Domżał,[†] Dirk Valkenborg,[‡] Wouter Maes,[§,‖,⊥] and Anna Gambin[*,†]

†*Faculty of Mathematics, Informatics and Mechanics, University of Warsaw, Banacha 2, Warsaw, 02-097, Poland*

‡*Data Science Institute, Hasselt University, Hasselt, 3500, Belgium*

¶*Department of Applied Biomedical Science, Faculty of Health Sciences, University of Malta, Msida, MSD 2080, Malta*

§*Institute for Materials Research (IMO), Hasselt University, Agoralaan, Diepenbeek, 3590, Belgium*

‖*IMEC, Associated lab IMOMEC, Wetenschapspark 1, Diepenbeek, 3590, Belgium*

⊥*Energyville, Thorpark, Genk, 3600, Belgium*

E-mail: a.gambin@uw.edu.pl

Supplementary Tables

	M_n (kg/mol)	M_w (kg/mol)	$Đ$
P1	22.6	38.4	1.7
P2	22.1	50.3	2.3
P3	11.7	56.0	4.8

Supplementary Table S1: Molar mass data for the different PBTTT samples.

Spectrum	Proportion threshold value	P1	P2	P3	$P3_{7p}$
Polymers detected	0	92	156	112	114
	0.002	57	90	67	68
Signal matched to polymer species (%)	0	93	88	90	90
	0.002	91	83	86	86
Signal not matched to polymer species (%)	0	7	12	10	10
	0.002	9	17	14	14

Supplementary Table S2: A summary of the Masserstein annotations. "Polymers detected" is the number of different polymer species of the form (#TT_i, #BT_i, E_i, p_i) such that their associated non-normalized proportion p_i is either greater than 0 or greater than 0.002 (the Proportion threshold value). "Signal matched to polymer species" is the percentage of the signal in the spectra P1-P3 and $P3_{7p}$ that was identified as corresponding to one of the detected polymer species. "Signal not matched to polymer species" is the percentage of signal assumed to come from the matrix, contaminants, non-polymer ions, or polymer species not included in the library. This value includes polymers below the proportion threshold. Setting a proportion threshold of 0.002 in order to limit the false positive annotations has the effect of discarding multiple polymer species, but has a minor influence on the percentage of explained signal.

Suplementary Figures

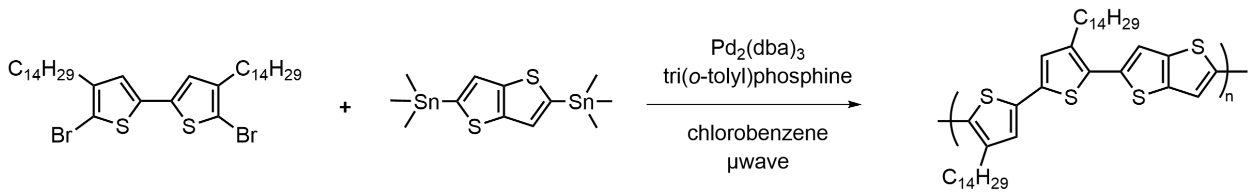

Supplementary Figure S1: PBTTT polymer synthesis.

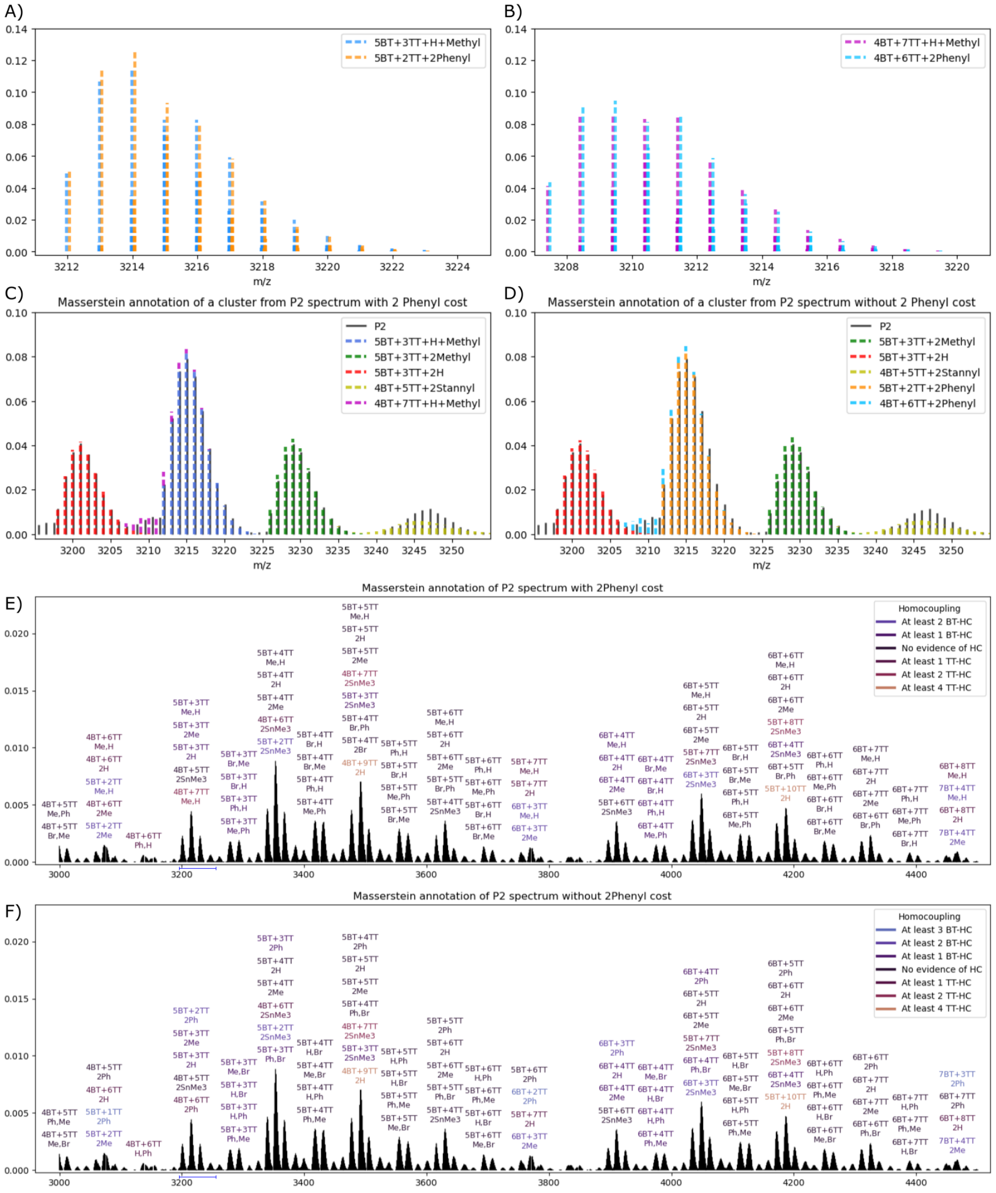

Supplementary Figure S2: Examples of annotation with and without adding the annotation penalty for polymers with two Phenyl end-groups. A, B) Polymer species nBT+mTT+H+Methyl and nTT+(m-1)TT+2Phenyl have nearly indistinguishable isotopic envelopes. Quantifying both polymers simultaneously from mass spectra is unreliable. C, D) Annotation with the (H, Methyl) polymers shows a clear pattern in which the (H, Methyl) polymer has two times the proportion of (H, H) and (Me, Me) polymers. Annotation with 2Phenyl doesn't show such a pattern, making this polymer the less likely one. E, F) Without the added penalty, Masserstein returns multiple annotations with 2Phenyl polymer chains in place of (H, Methyl). This shows that in the presence of measurement errors, isotopic envelopes are insufficient to distinguish between these compounds, and the less likely one may by chance fit the data better. This motivates introducing an additional penalty for less probable compounds so that the software annotates them only if the experimental evidence is sufficiently robust.

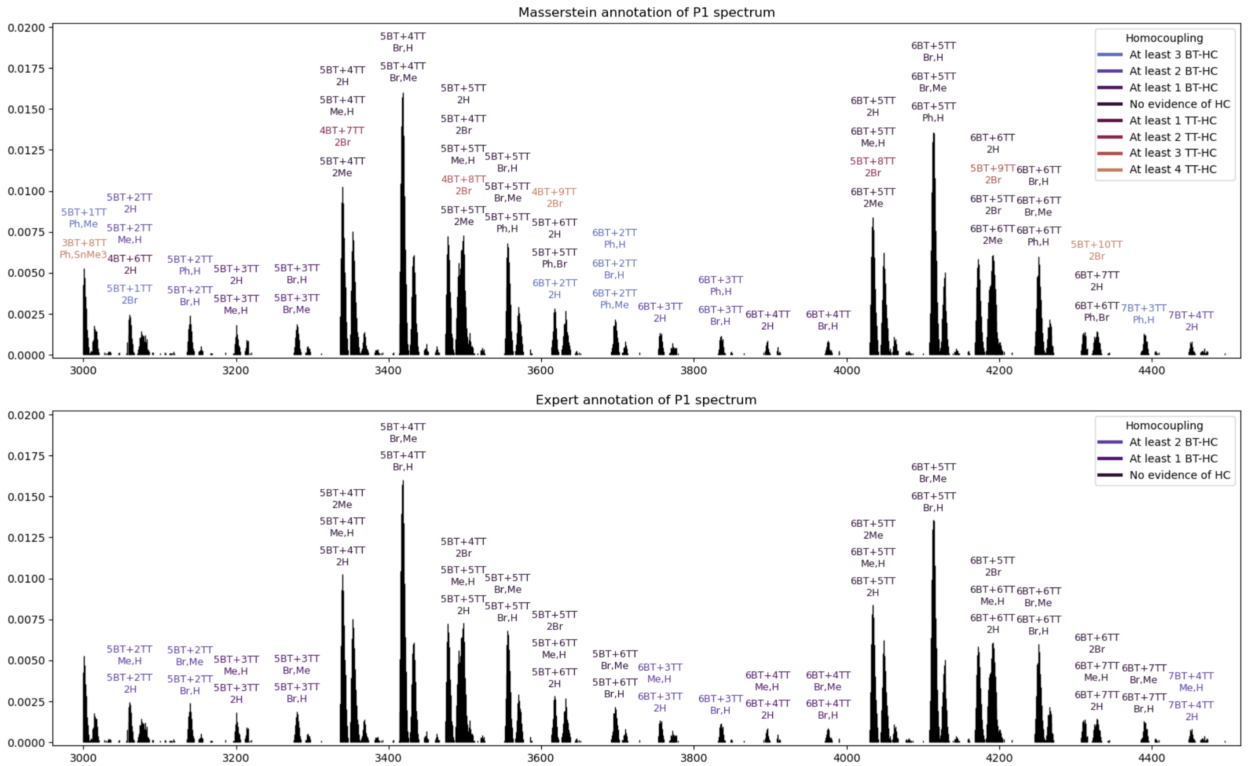

Supplementary Figure S3: A comparison of annotations generated automatically by Masserstein (top) and manually by an expert (bottom) for spectrum P1.

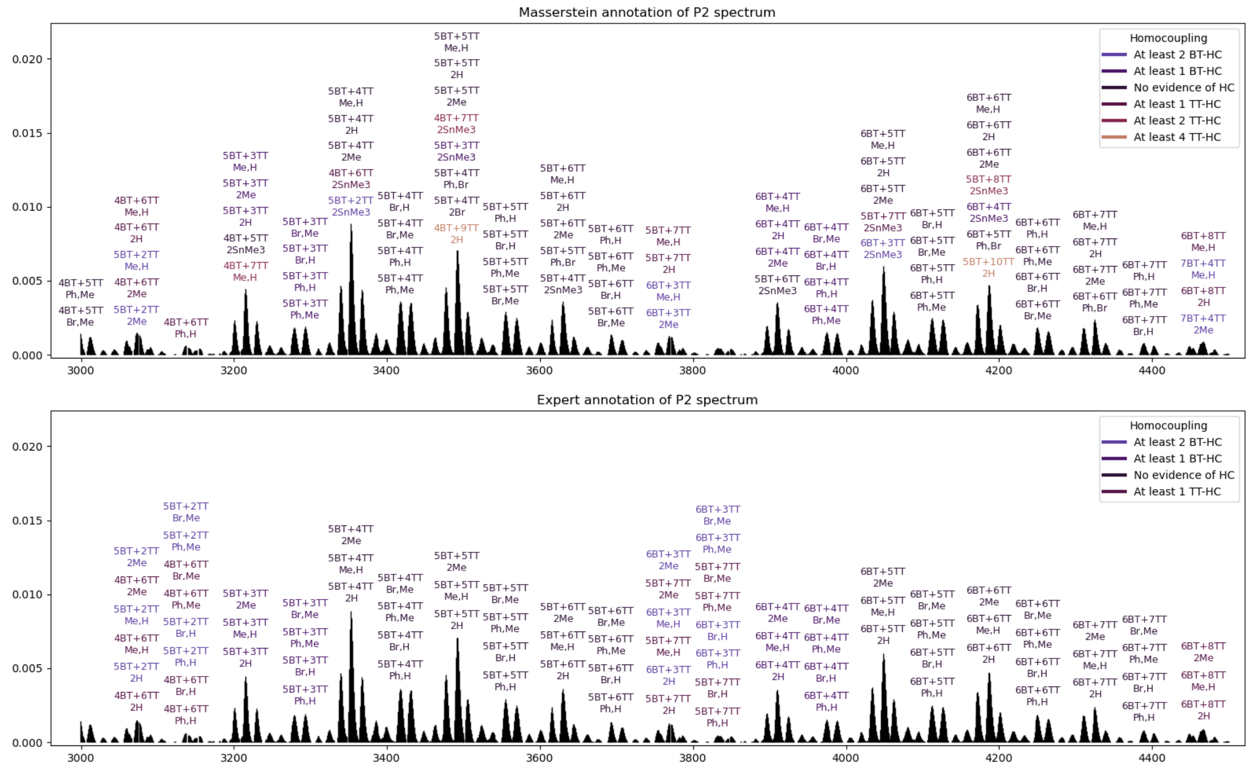

Supplementary Figure S4: A comparison of annotations generated automatically by Masserstein (top) and manually by an expert (bottom) for spectrum P2.

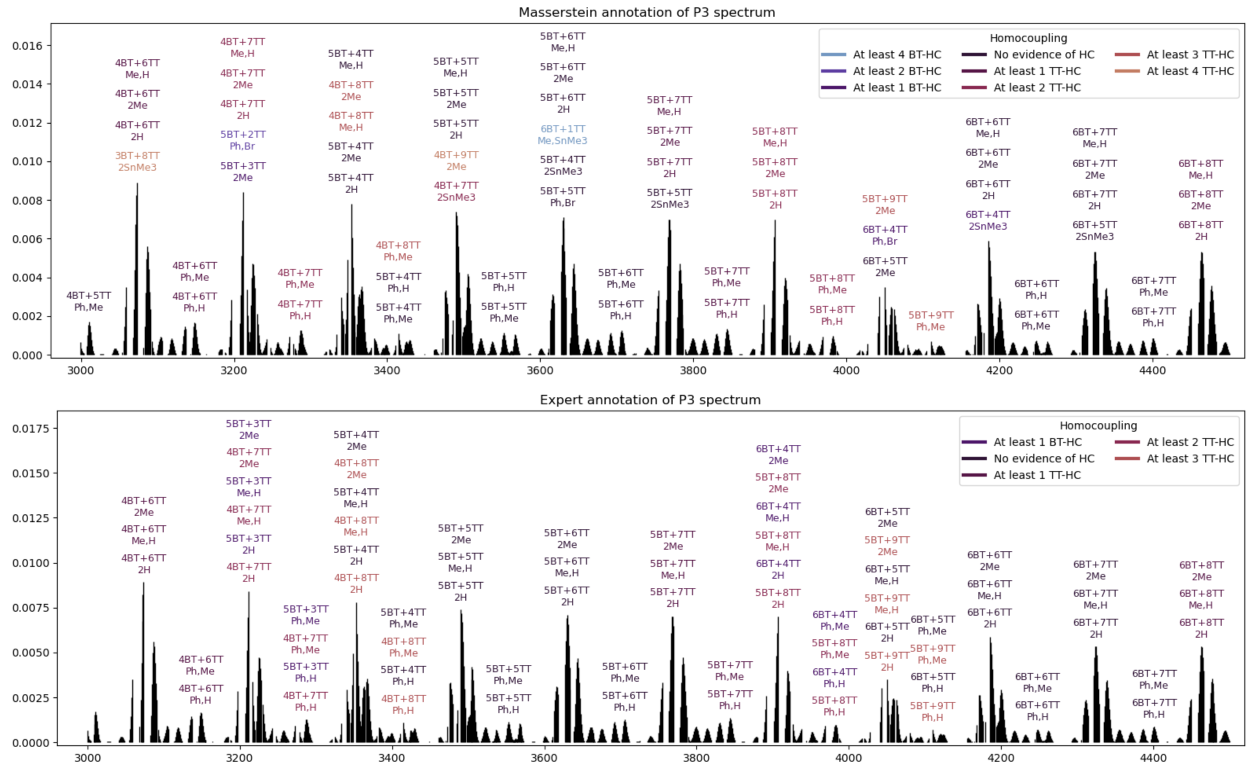

Supplementary Figure S5: A comparison of annotations generated automatically by Masserstein (top) and manually by an expert (bottom) for spectrum P3.

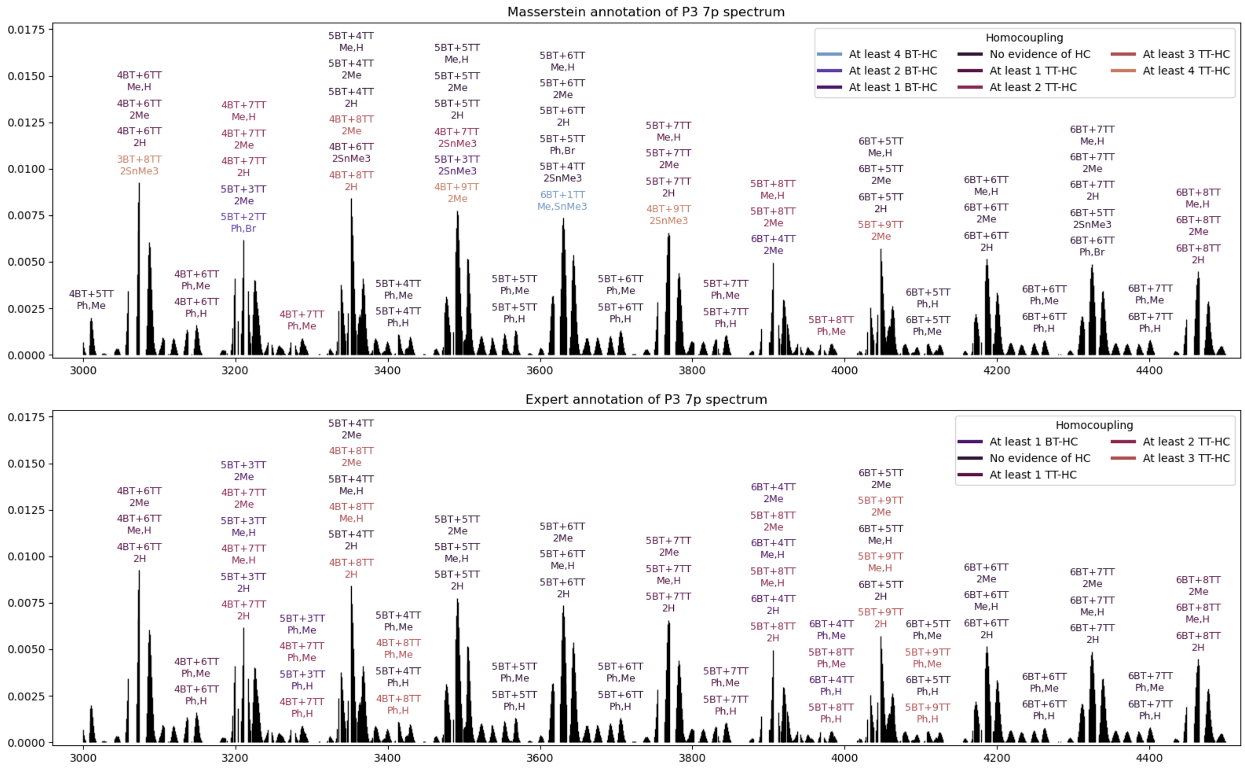

Supplementary Figure S6: A comparison of annotations generated automatically by Masserstein (top) and manually by an expert (bottom) for spectrum $P3_{7p}$.

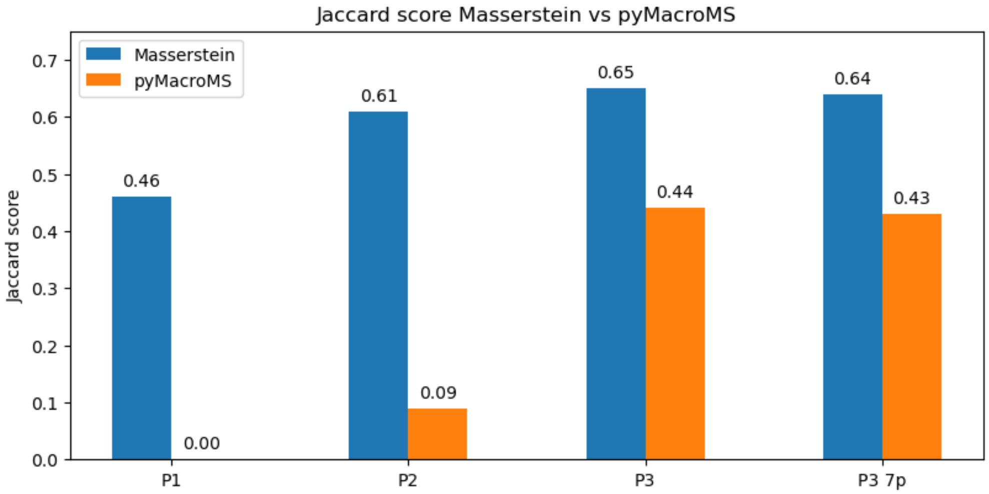

Supplementary Figure S7: The Jaccard score of annotations obtained with Masserstein and pyMacroMS.

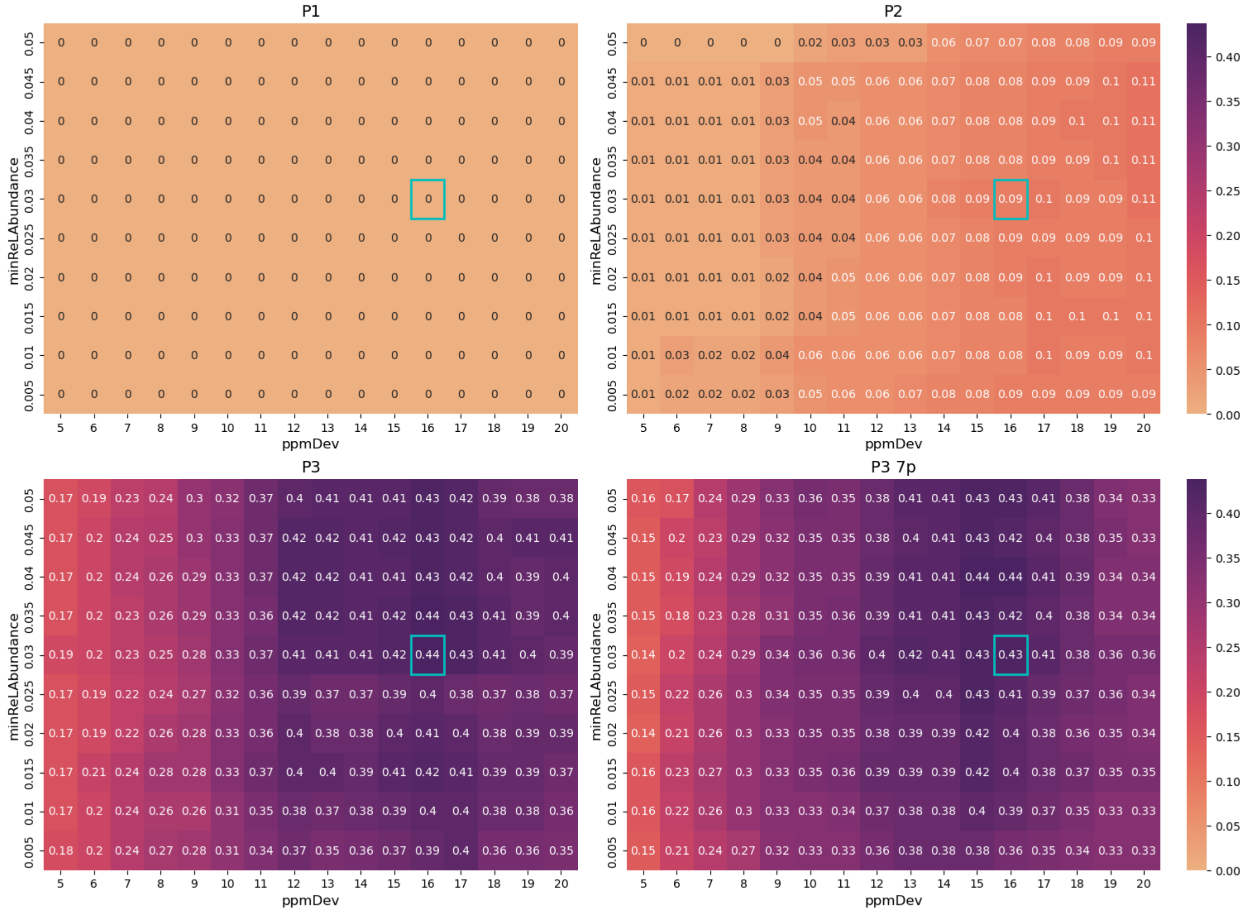

Supplementary Figure S8: The Jaccard score of annotations obtained with pyMacromMS depending on `minRelAbundance` and `ppmDev` parameters.

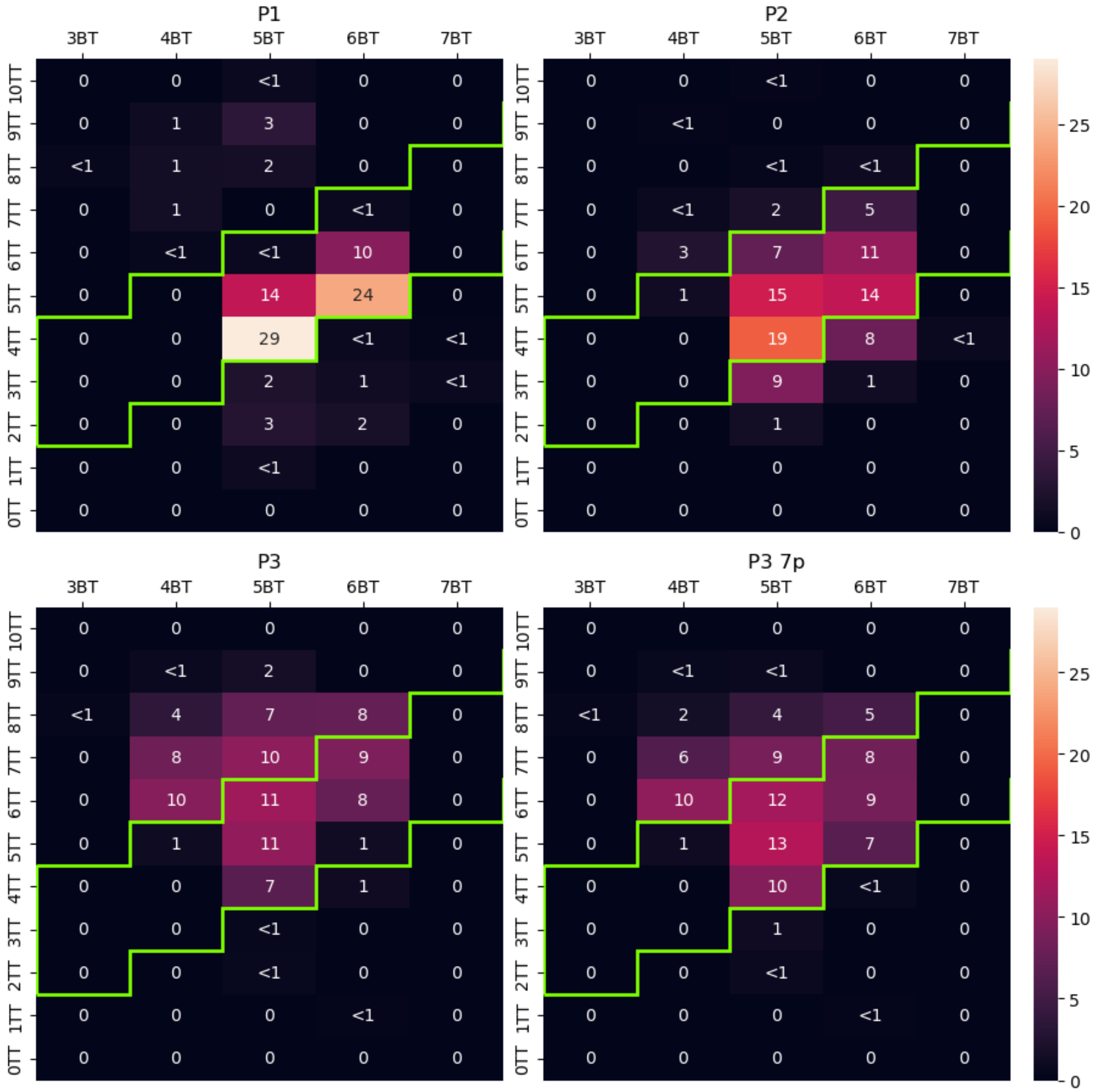

Supplementary Figure S9: Estimated percentages of polymers in experimental samples by Masserstein summed up by monomer counts (rounded to integer values). Estimated percentages were normalized so the explained signal equals 100%. The diagonal lines delineate the regions with evidence for TT homocoupling (top-left corners) and BT homocoupling (bottom-right corners).

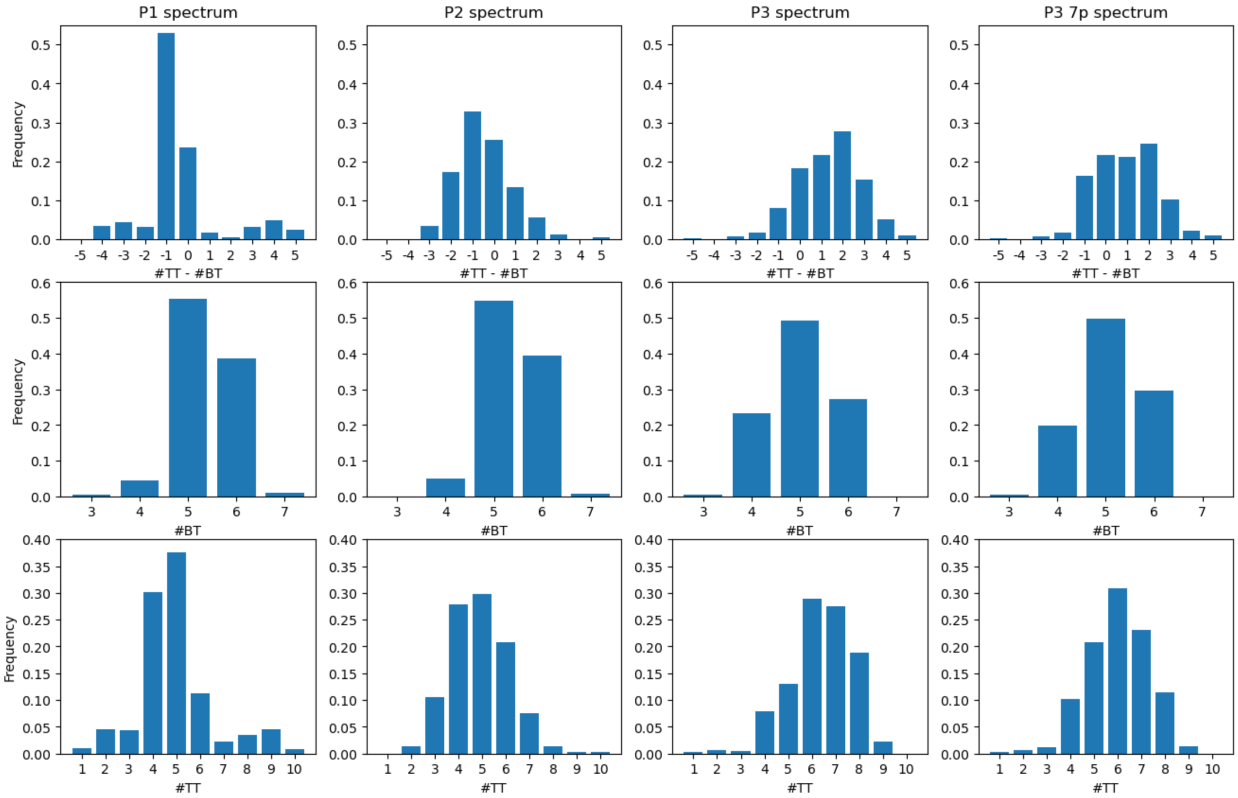

Supplementary Figure S10: The distributions of the difference of subunit counts $\Delta = \#TT - \#BT$, the number of BT subunits, and the number of TT subunits in polymer species annotated with Masserstein in spectra of PBTTT. In spectrum P1, the distribution of Δ shows an unexpected sub-population of heavily TT-homocoupled polymer chains which may be an artifact of the software annotation.

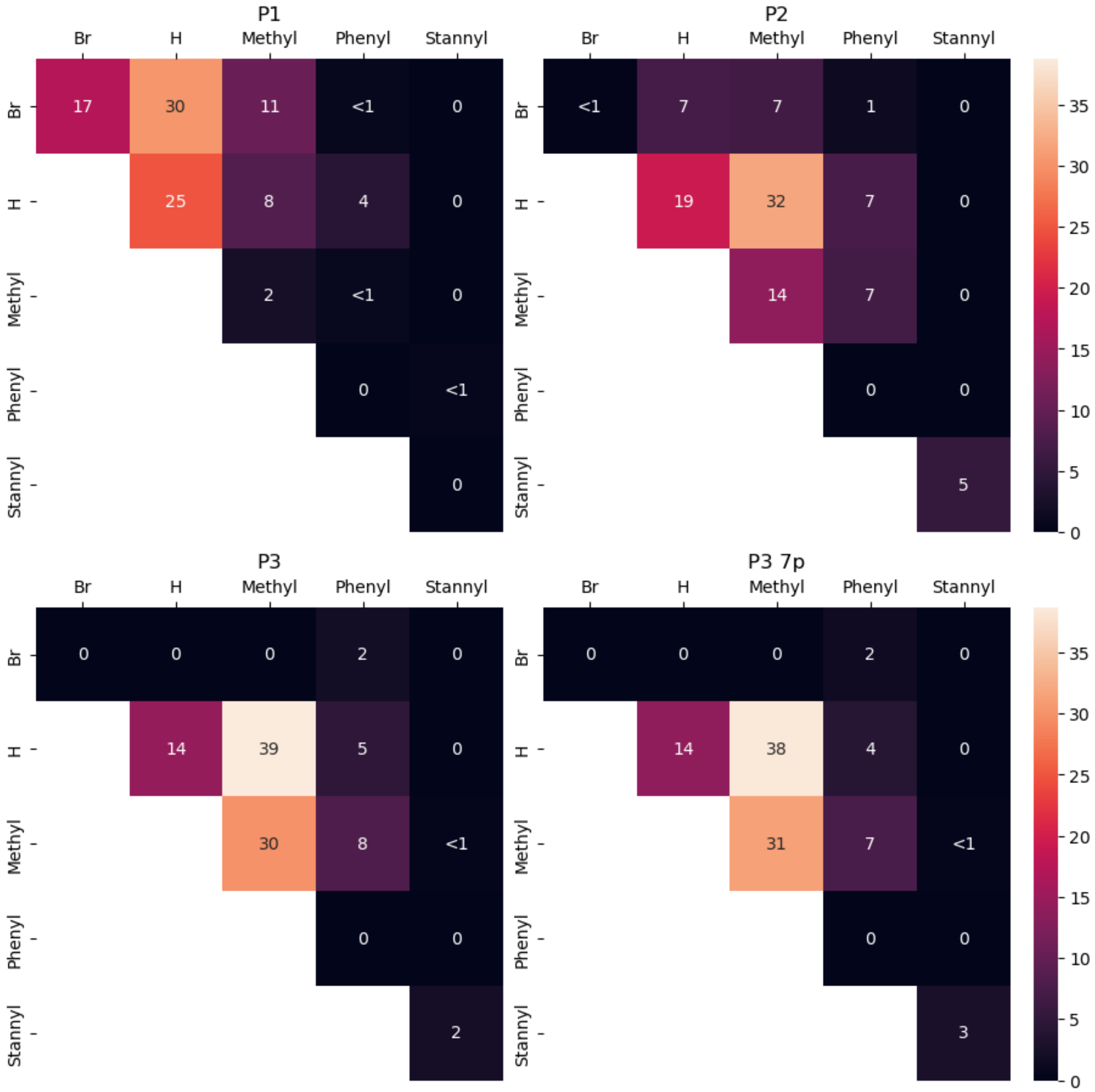

Supplementary Figure S11: Estimated percentages of polymers in experimental samples by Masserstein summed up by polymer end-groups, rounded to integer values. Estimated percentages were normalized so the explained signal equals 100%.

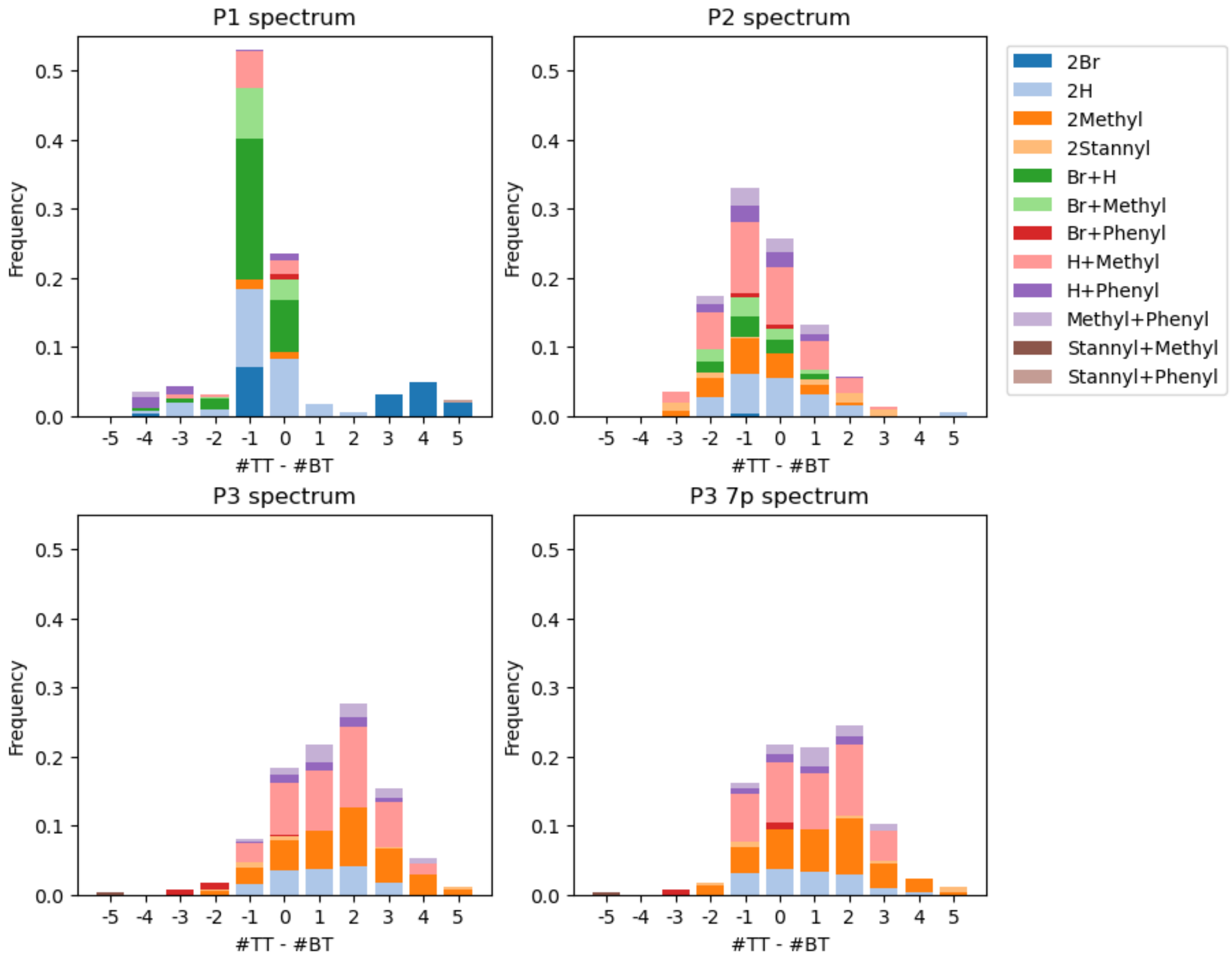

Supplementary Figure S12: The distribution of the difference of subunit counts, $\Delta = \#TT - \#BT$, in the polymers annotated with Masserstein. The Y axis corresponds to the estimated proportion of signal corresponding to polymer species with a given value of Δ. The bars are colored according to the contribution of polymer species with each end-group composition.

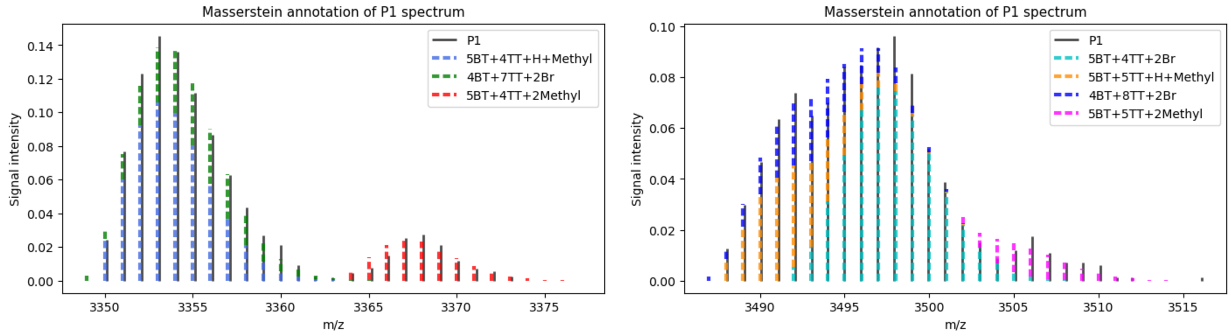

Supplementary Figure S13: Examples of Masserstein deconvolution of clusters from the P1 spectrum consisting of heavily TT-homocoupled polymer species with 2 Br end-groups: 4BT+7TT+2Br and 4BT+8TT+2Br. Such species are chemically not plausible, because the bromine end-group is associated with the BT subunits. Consequently, this chemical composition would suggest polymer chains with BT subunits at the ends and blocks of TT subunits in the middle, which are unlikely (albeit not impossible) to be produced by the Stille condensation reaction. However, the isotopic envelopes of these species seem to agree well with the data, and our library of reference spectra does not offer an alternative explanation of these signals. The result therefore seems both statistically supported and chemically implausible.

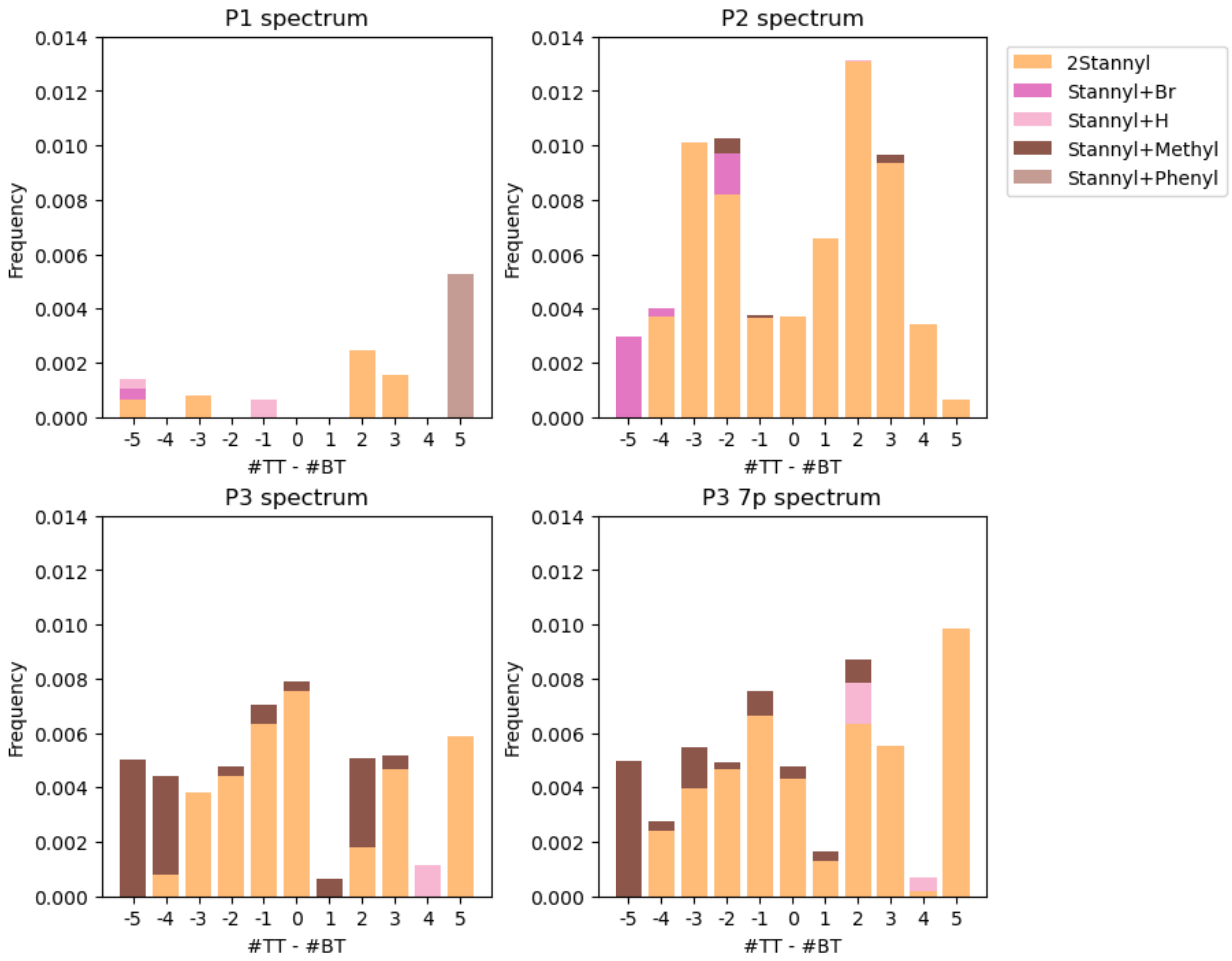

Supplementary Figure S14: The distribution of the difference of subunit counts, $\Delta = \#TT - \#BT$, for polymer chains with one or two stannyl end-groups, including the species below the proportion threshold of 0.002. The stannyl end-groups are associated with the TT subunit, and therefore were not expected to be found in heavily BT-homocoupled polymer chains, suggesting an artifactual annotation. However, the distribution including the polymers below the threshold reveals that low values of the Δ statistic are associated with polymers chains with one, rather than two, stannyl end-groups, and that increasing values of this statistic are associated with increasing proportions of polymer chains with two stannyl end-groups. This suggests that these are *bona fide* annotations and that the analyzed samples do contain low amounts of heavily BT-homocoupled polymer chains with a stannyl end-group at one end.

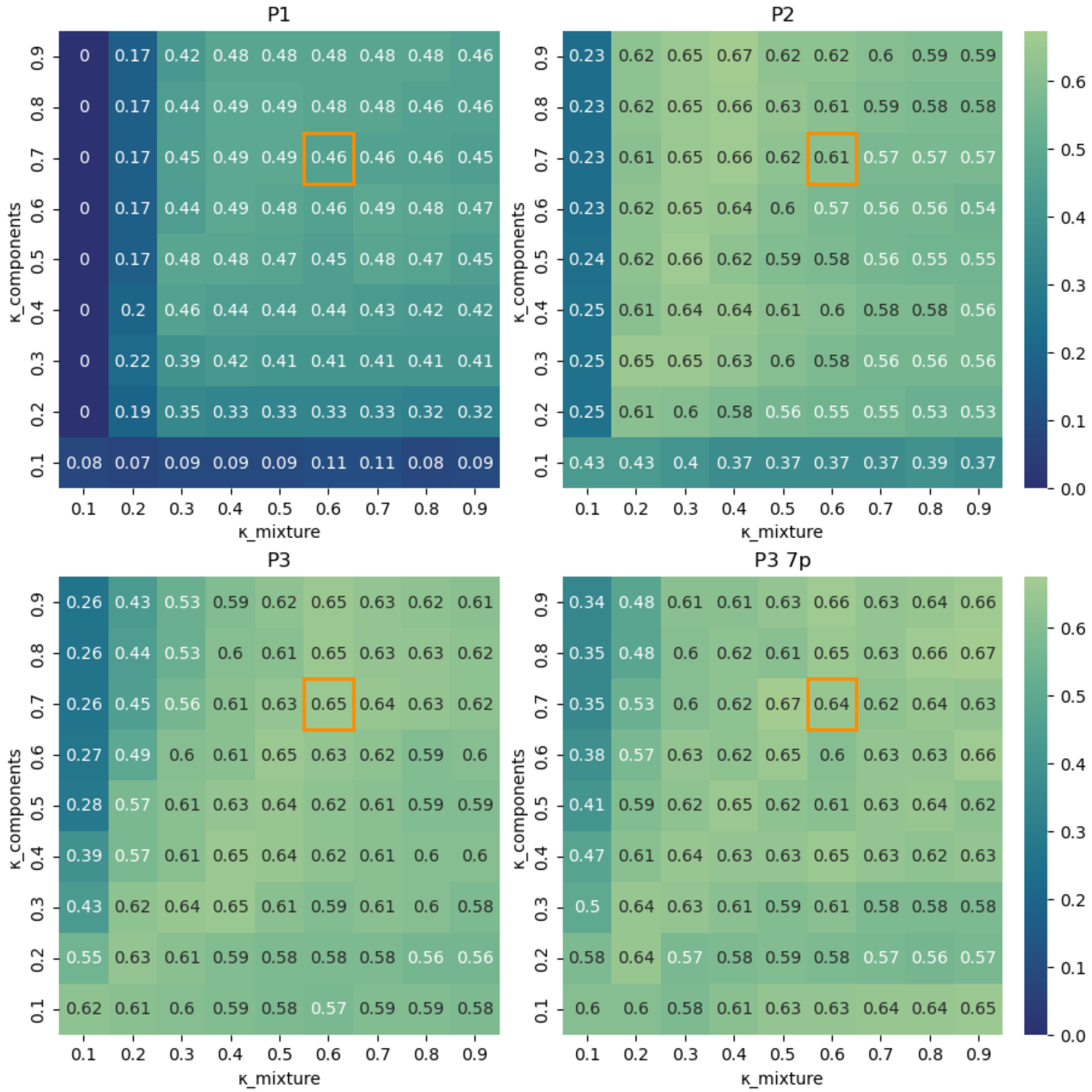

Supplementary Figure S15: Jaccard similarity score depending on different κ_{mixture} and $\kappa_{\text{components}}$ values. While the annotation accuracy is stable for moderate changes of κ_{mixture} and $\kappa_{\text{components}}$, gross misspecification of these parameters leads to incorrect results. The highlighted squares correspond to parameter values selected for the statistical analysis of homocoupling patterns. Notably, these values yield accurate results in all of the experimental spectra, indicating that a single parameter tuning can be used for analyses of multiple spectra. Moreover, these values were selected based on a manual inspection of the fitted model in a few selected regions of the spectra P1 and P2, without the need for a manual annotation.

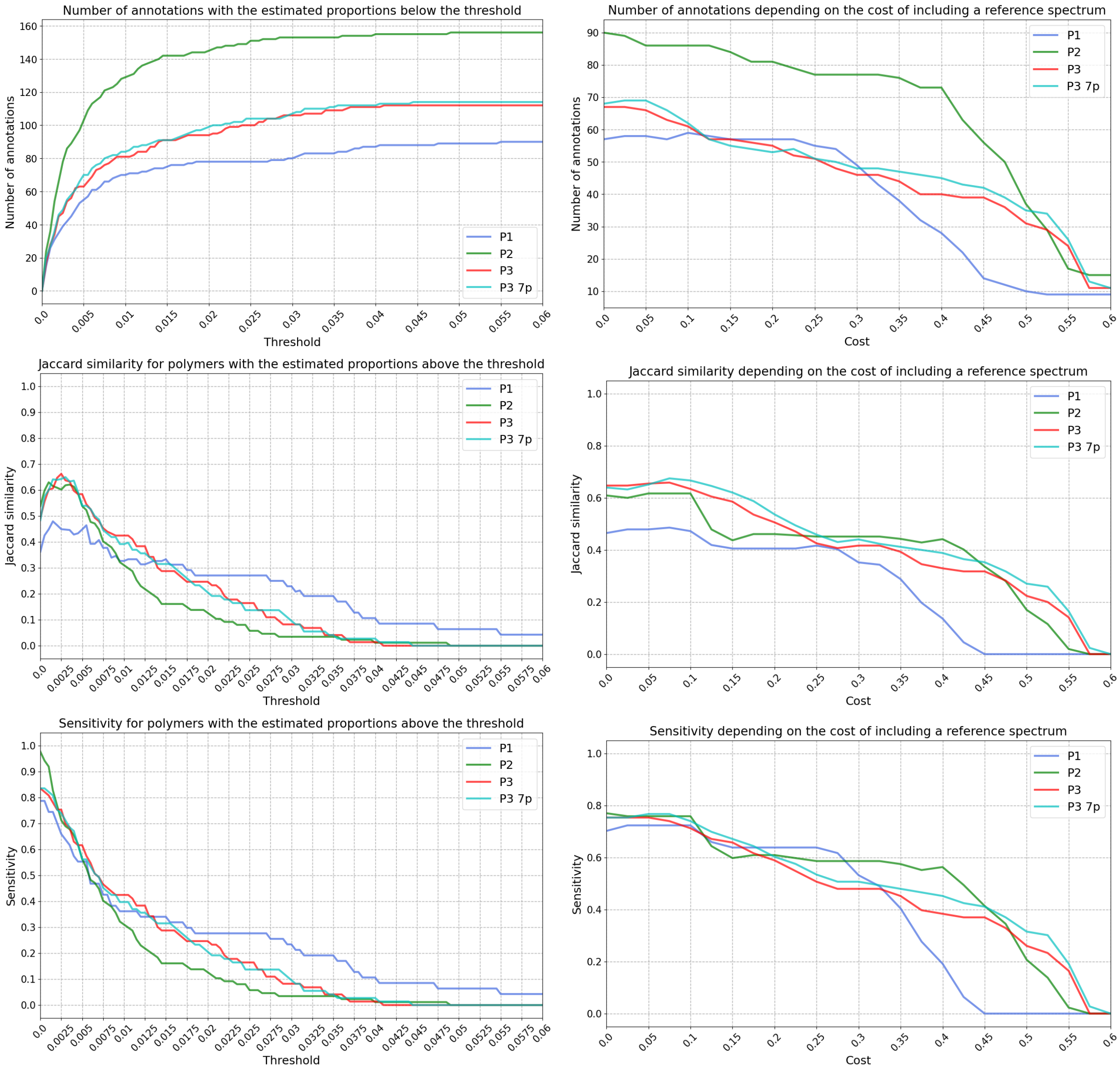

Supplementary Figure S16: Left column: The effect of proportion threshold on the automatic annotation results of experimental spectra. The molecular species with estimated proportions below the threshold were discarded. Right column: The effect of annotation penalty (right column) on the automatic annotation results of experimental spectra. Only the penalty for polymer species other than those with Ph-Ph end-groups varied. First row: The number of discovered polymers. Second row: The Jaccard similarity between the Masserstein and the expert annotations. Third row: The sensitivity of the Masserstein annotation (defined as the number of identical annotations divided by the number of expert annotations).

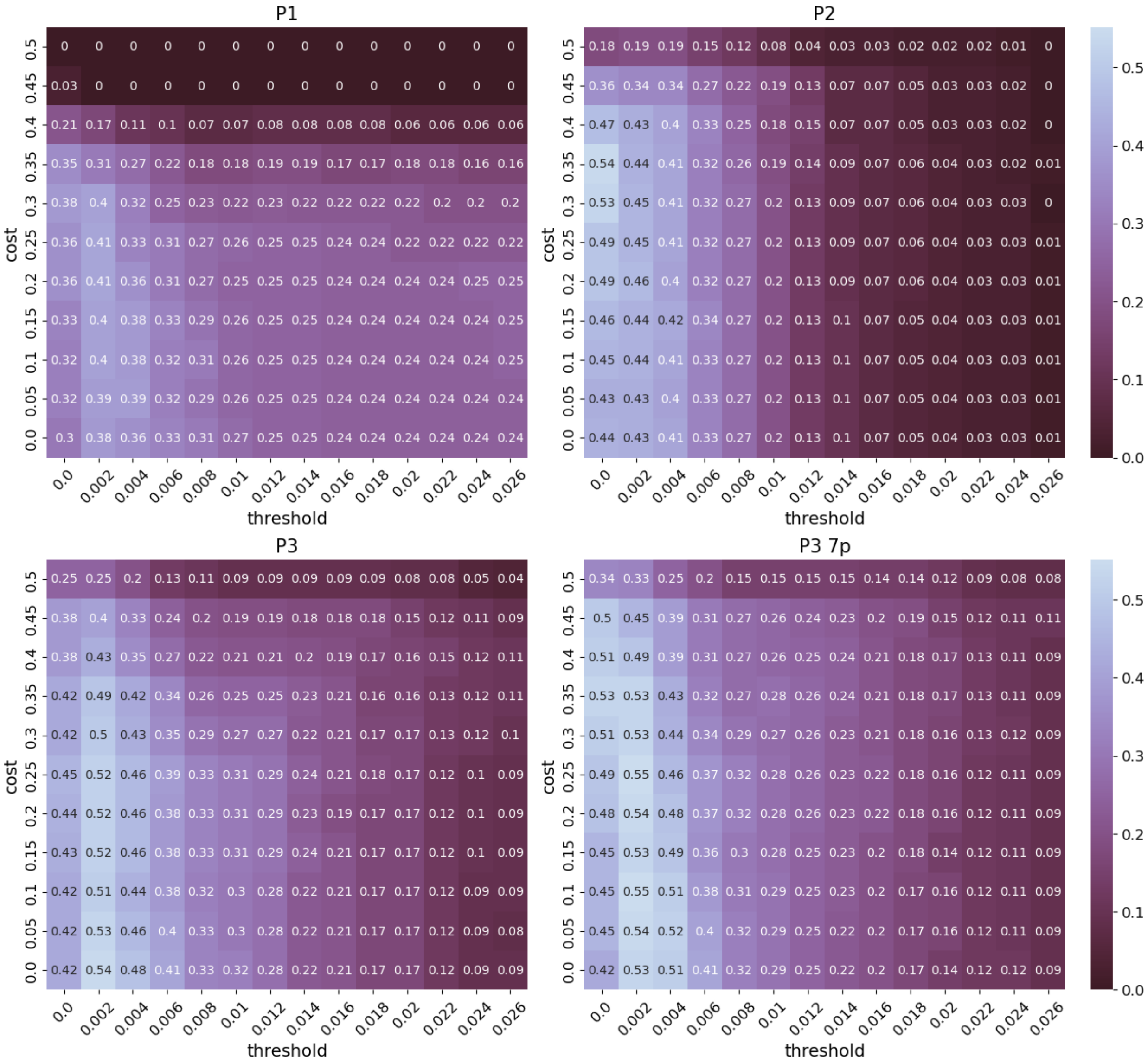

Supplementary Figure S17: The Jaccard score similarity between the Masserstein and the expert annotations depending on the cost of including a reference spectrum in the deconvolution (parameter c) and the threshold of the estimated proportion below which polymer species are discarded.

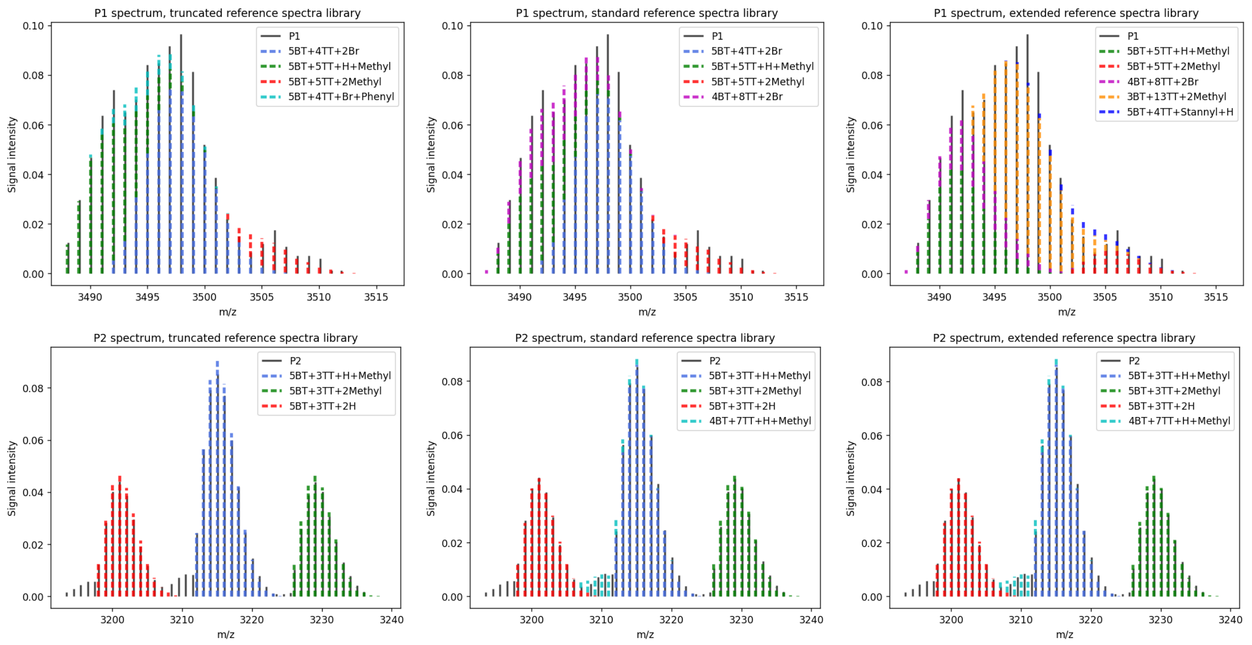

Supplementary Figure S18: The impact of the selection of the library of reference spectra on the annotation of polymer spectra. Left column: "truncated" library (max difference between monomer counts in a polymer chain = 2; 163 spectra); Middle column: "intermediate" library (max difference = 5; 359 spectra); Right column: "extended" library (max difference = 10; 570 spectra). Using the extended library allows for discovering more compounds, but also results in spurious annotations with heavily homocoupled compounds (e.g. 3BT+13TT+2Methyl in the top row). Such annotations may result in a gross overestimation of homocoupling defects. On the other hand, while using the truncated library limits the number of false annotations, it fails to detect some compounds (e.g. 4BT+7TT+2H in the middle row). This may, in turn, result in an underestimation of the structural defects and bias the analysis. The intermediate library, which we used for statistical analyses in this work, seems to balance the two factors. However, it does not fully eliminate the risk of spurious annotations, which is one of the possible reasons for the sub-population of heavily homocoupled polymer chains for P1 in Supplementary Fig. S10. This points to the fact that the results of automatic annotation are sensitive to the selection of the library of reference spectra, and further research is needed to optimize the libraries and to develop more robust algorithms.

The Wasserstein distance

Below, we introduce the notion of the Wasserstein distance. We only describe a one-dimensional case here, as it is sufficient for the purposes of this paper.

Definition 1. *Let $X \subset \mathbb{R}$ and let $\mathcal{P}(X)$ be the space of probability measures defined on X. Let's define $\mathcal{P}_1(X)$ as:*

$$\mathcal{P}_1(X) := \{\mu \in \mathcal{P}(X) : \int_X |x| d\mu(x) < +\infty\}.$$

Definition 2. *Let $\mu \in \mathcal{P}_1(X)$, $\nu \in \mathcal{P}_1(Y)$. A probability measure $\gamma : X \times Y \to [0, 1]$ satisfying conditions:*

$$\int_{y \in Y} d\gamma(x, y) = \mu(x), \qquad \int_{x \in X} d\gamma(x, y) = \nu(y)$$

is called a transport plan between μ and ν. We denote a set of all transport plans between μ and ν with $\Gamma = \Gamma(\mu, \nu)$.

Definition 3. *Let μ, $\nu \in \mathcal{P}_1(X)$. We define the Wasserstein distance (the Wasserstein metric) between μ and ν as*

$$W_{|.|}(\mu, \nu) = \min_{\gamma \in \Gamma(\mu, \nu)} \int_{X \times X} |x - y| d\gamma(x, y).$$

In a more general formulation, the absolute value in the above definition can be replaced by some other distance function $\rho : X \times X \to [0, +\infty)$. Then we have:

$$W_\rho(\mu, \nu) = \min_{\gamma \in \Gamma(\mu, \nu)} \int_{X \times X} \rho(x, y) d\gamma(x, y).$$

Proof of the main theoretical result

The main goal of the estimation is to find proportions $p_0'^*, p_0^*, p^*$ such that

$$p_0'^*, p_0^*, p^* = \underset{p_0', p_0, p=(p_1,\dots,p_k)}{\arg\min} \{W\left((1-p_0')\mu + p_0'\xi, \nu_p + p_0\omega\right) + c^T p\}, \tag{1}$$

where ξ is a new auxiliary point corresponding to noise in reference spectra, μ is an experimental spectrum, $\nu_p = \sum_{j=1}^{k} p_j \nu_j$ is a linear combination of reference spectra $\nu_1, \dots, \nu_k$, and ω is an auxiliary point corresponding to noise in the experimental spectrum. Moreover $c^T p = \sum_{j=1}^{k} c_j p_j$ is a linear combination of penalties of reference spectra $c_1, \dots, c_k$, where c_j is the cost of using ν_j to explain experimental signal.

Let's note that for $p_0', p_0, p_1, ..., p_k$ we have

$$p_0 + \sum_{i=1}^{k} p_i = 1 \tag{2}$$

$$0 \leq p_0', p_0, p_1, ..., p_k \leq 1.$$

Furthermore, we assume that the experimental spectrum and the reference spectra are normalized, i.e.:

$$\int_{x \in \mathbb{R}} d\mu(x) = 1,$$
$$\int_{y \in \mathbb{R}} d\nu_j(y) = 1 \text{ for } j = 1, 2, ..., k.$$

The reason why we multiply μ by $(1 - p_0')$ here is that we assume that some fraction of μ is invisible to us. There exists some amount of signal in the combination of reference spectra that is absent in the experimental spectrum μ. Since μ itself is normalized we treat it as incomplete, we have to rescale it, so there is p_0' of signal left for the hidden part.

Additionally, we need to assume that

$$1 - p_0 - p_0' \geq 0. \quad (3)$$

This assumption guarantees that the amount of signal removed to ω is not greater than the amount of signal present in the rescaled experimental spectrum (i.e. $1 - p_0' \geq p_0$) and the amount of signal removed to ξ is not greater than the amount of signal present in reference spectra (i.e. $1 - p_0 \geq p_0'$).

Let's denote by $M(t)$ and $N_p(t) = \sum_{j=1}^{k} p_j N_j(t)$ cumulative distribution functions of μ and ν_p, respectively.

We are going to prove the equivalence of two formulations of Problem 1, but first let's recall a useful Theorem proved in.[1]

Theorem 1. *Let μ and ν be two probability measures on the real line $\mathbb{R}$. Let M and N be the cumulative distribution function of μ and ν respectively. Then*

$$W(\mu, \nu) = \int_{\mathbb{R}} |M(t) - N(t)| dt. \quad (4)$$

The following Theorem provides a more computationally convenient way of finding optimal proportions and was first formulated and proven in.[2] Since the previous version does not incorporate penalties into the minimization problem, we present an updated formulation.

Theorem 2. *Problems of finding*

$$p_0'^{*}, p_0^{*}, p^{*} = \underset{p_0', p_0, p}{\arg\min} \{ W\left((1 - p_0')\mu + p_0'\xi, \nu_p + p_0\omega\right) + c^T p \}$$

and

$$p_0'^*, p_0^*, p^*, g^*, g'^* =$$

$$= \underset{p_0', p_0, p, g, g'}{\arg\min} \{\kappa p_0 + \kappa' p_0' + c^T p + \sum_{i=1}^{n-1} (s_{i+1} - s_i) \left|(1 - p_0')M(s_i) + G'(s_i) - G(s_i) - N_p(s_i)\right|\}$$

are equivalent.

As mentioned, the version of Theorem 2 without penalties is proved in,[2] however, for the convenience of the reader we will quote it below.

Proof. First, let's define the distance function as follows:

$$\rho(x, y) = \begin{cases} |x - y| & \text{if } x, y \in \mathbb{R}, \\ \kappa & \text{if } x \in \mathbb{R}, y = \omega, \\ \kappa' & \text{if } x = \xi, y \in \mathbb{R}, \\ \kappa + \kappa' & \text{if } x = \xi, y = \omega. \end{cases}$$

Therefore, the Wasserstein distance between $(1 - p_0')\mu + p_0'\xi$ and $\nu_p + p_0\omega$ can be expressed as:

$$\begin{aligned}
& W((1 - p_0')\mu + p_0'\xi, p_0\omega + p_1\nu_1 + \cdots + p_k\nu_k) = \\
& = \min_{\gamma \in \Gamma} \int_{x \in \mathbb{R} \cup \{\xi\}} \int_{y \in \mathbb{R} \cup \{\omega\}} \rho(x, y) d\gamma(x, y) \\
& = \min_{\gamma \in \Gamma} \left(\int_{x \in \mathbb{R}} \int_{y \in \mathbb{R}} |x - y| d\gamma(x, y) \int_{x \in \mathbb{R}} \kappa d\gamma(x, \omega) + \int_{y \in \mathbb{R}} \kappa' d\gamma(\xi, y) + (\kappa + \kappa') \right) \\
& = \min_{\gamma \in \Gamma} \left(\int_{x \in \mathbb{R}} \int_{y \in \mathbb{R}} |x - y| d\gamma(x, y) + \kappa(p_0 - \gamma(\xi, \omega)) + \kappa'(p_0' - \gamma(\xi, \omega)) + (\kappa + \kappa')\gamma(\xi, \omega) \right) \\
& = \min_{\gamma \in \Gamma} \left(\kappa p_0 + \kappa' p_0' + \int_{x \in \mathbb{R}} \int_{y \in \mathbb{R}} |x - y| \gamma(x, y) dy dx \right) \\
& = \kappa p_0 + \kappa' p_0' + \min_{\gamma \in \Gamma} \int_{x \in \mathbb{R}} \int_{y \in \mathbb{R}} |x - y| \gamma(x, y) dy dx.
\end{aligned}$$

To be able to use Theorem 1 we need to be working with probabilistic measures defined on $\mathbb{R}^2$. That is currently not the case, so let's define two measures: $g(x) = \gamma(x, \omega)$ and $g'(y) = \gamma(\xi, y)$ with $G(t)$ and $G'(t)$ being their cumulative distribution functions. Note that for all $x \in \mathbb{R}$

$$\int_{x\in\mathbb{R}} \gamma(x,y)dx = p_1\nu_1 + \cdots + p_k\nu_k - \gamma(\xi, y)$$

$$= p_1\nu_1 + \cdots + p_k\nu_k - g'(y) \quad \text{if } y \in \mathbb{R}, \tag{5}$$

$$\int_{x\in\mathbb{R}} \gamma(x,\omega)dx = \int_{x\in\mathbb{R}} dg(x)$$

$$= p_0 - \gamma(\xi, \omega), \tag{6}$$

$$\int_{y\in\mathbb{R}} \gamma(x,y)dy = (1 - p_0')\mu(x) - \gamma(x, \omega)$$

$$= (1 - p_0')\mu(x) - g(x) \tag{7}$$

$$\int_{y\in\mathbb{R}} \gamma(\xi,y)dy = \int_{y\in\mathbb{R}} dg'(y)$$

$$= p_0' - \gamma(\xi, \omega), \tag{8}$$

$$\int_{x\in\mathbb{R}}\int_{y\in\mathbb{R}} \gamma(x,y)dydx = (1 - p_0')\mu(x) - \gamma(x, \omega)$$

$$= 1 - p_0' - p_0 + \gamma(\xi, \omega) \tag{9}$$

Moreover, from the assumption described by Eq. (3), we infer that

$$1 - p_0 - p_0' + \gamma(\xi, \omega) \geq 0. \tag{10}$$

Now, let's show that $\gamma_{|\mathbb{R}^2}(x, y)//(1 - p_0 - p_0' + \gamma(\xi, \omega))$ is a probabilistic measure. Indeed,

we have:

$$\int_{x\in\mathbb{R}}\int_{y\in\mathbb{R}} \gamma_{|\mathbb{R}^2}(x,y)//(1-p_0-p_0'+\gamma(\xi,\omega))dydx = \frac{\int_{x\in\mathbb{R}}(1-p_0')\mu(x)-g(x)}{1-p_0-p_0'+\gamma(\xi,\omega)} \tag{11}$$

$$= \frac{1-p_0'-p_0+\gamma(\xi,\omega)}{1-p_0-p_0'+\gamma(\xi,\omega)} \tag{12}$$

$$= 1,$$

$$\int_{y\in\mathbb{R}}\int_{x\in\mathbb{R}} \gamma_{|\mathbb{R}^2}(x,y)//(1-p_0-p_0'+\gamma(\xi,\omega))dxdy = \frac{\int_{x\in\mathbb{R}} p_1\nu_1+\cdots+p_k\nu_k-g'(y)}{1-p_0-p_0'+\gamma(\xi,\omega)} \tag{13}$$

$$= \frac{\sum_{j=1}^{k} p_j - p_0' + \gamma(\xi,\omega)}{1-p_0-p_0'+\gamma(\xi,\omega)} \tag{14}$$

$$= \frac{1-p_0-p_0'+\gamma(\xi,\omega)}{1-p_0-p_0'+\gamma(\xi,\omega)}$$

$$= 1,$$

where Eq. (11) and Eq. (13) follow from Eq. (7) and Eq. (5) respectively, Eq. (12) and Eq. (14) follow from Eq. (6) and Eq. (8) as well as the fact that μ and ν_j for $j = 1,\ldots,k$ are probability measures. So, consequently $\gamma_{|\mathbb{R}^2}(x,y)//(1-p_0-p_0'+\gamma(\xi,\omega))$ is in fact probability measure.

Next, we will find marginal distributions of $\gamma_{|\mathbb{R}^2}(x,y)//(1-p_0-p_0'+\gamma(\xi,\omega))$ that will be needed in the subsequent part of the proof. We have:

$$\int_{y\in\mathbb{R}} \frac{\gamma_{|\mathbb{R}^2}(x,y)}{1-p_0-p_0'+\gamma(\xi,\omega)}dy = \frac{(1-p_0')\mu(x)-g(x)}{1-p_0-p_0'+\gamma(\xi,\omega)} \tag{15}$$

$$\int_{x\in\mathbb{R}} \frac{\gamma_{|\mathbb{R}^2}(x,y)}{1-p_0-p_0'+\gamma(\xi,\omega)}dx = \frac{p_1\nu_1+\cdots+p_k\nu_k-g'(y)}{1-p_0-p_0'+\gamma(\xi,\omega)} \tag{16}$$

Knowing that $M(t), N(t), G(t), G'(t)$ are cumulative distribution functions of μ, ν, g, g' respectively, we can compute compute cumulative distribution function of the marginals

Eq. (15) and Eq. (16):

$$\int_{-\infty}^{t} \frac{(1-p_0')\mu(x) - g(x)}{1-p_0-p_0'+\gamma(\xi,\omega)} dx = \frac{(1-p_0')M(t) - G(t)}{1-p_0-p_0'+\gamma(\xi,\omega)}, \tag{17}$$

$$\int_{-\infty}^{t} \frac{\nu_p - g'(y)}{1-p_0-p_0'+\gamma(\xi,\omega)} = \frac{N(t) - G'(t)}{1-p_0-p_0'+\gamma(\xi,\omega)} \tag{18}$$

Now, we can apply Theorem 1 to our problem. To do this we firstly split minimization over all possible transport plans γ into minimization over $\gamma_{|\mathbb{R}^2}$ and over $g, g', \gamma(\xi,\omega)$:

$$\begin{aligned}
&\min_{\gamma\in\Gamma} \int_{x\in\mathbb{R}} \int_{y\in\mathbb{R}} |x-y|\gamma(x,y)dydx = \\
&= \min_{g,g',\gamma(\xi,\omega)} \min_{\gamma_{|\mathbb{R}^2}} \int_{x\in\mathbb{R}} \int_{y\in\mathbb{R}} |x-y|\gamma(x,y)dydx \\
&= \min_{g,g',\gamma(\xi,\omega)} (1-p_0-p_0'+\gamma(\xi,\omega)) \min_{\gamma_{|\mathbb{R}^2}} \int_{x\in\mathbb{R}} \int_{y\in\mathbb{R}} \frac{|x-y|\gamma(x,y)}{1-p_0-p_0'+\gamma(\xi,\omega)} dydx \\
&= \min_{g,g',\gamma(\xi,\omega)} (1-p_0-p_0'+\gamma(\xi,\omega)) \int_{t\in\mathbb{R}} \left| \frac{(1-p_0')M(t)-G(t)}{1-p_0-p_0'+\gamma(\xi,\omega)} - \frac{N(t)-G'(t)}{1-p_0-p_0'+\gamma(\xi,\omega)} \right| dt \\
&= \min_{g,g',\gamma(\xi,\omega)} \int_{t\in\mathbb{R}} |(1-p_0')M(t) - G(t) - N(t) + G'(t)|\, dt \\
&= \min_{g,g'} \int_{t\in\mathbb{R}} |(1-p_0')M(t) - G(t) - N(t) + G'(t)|\, dt,
\end{aligned}$$

where the third equality is a consequence of Theorem 1, the fact that $\gamma_{|\mathbb{R}^2}(x,y)//(1-p_0-p_0'+\gamma(\xi,\omega))$ is a probabilistic measure and equations Eq (17) and Eq. (18). In the fourth equality, we use the Eq. (10). The last equality holds because the minimization expression does not depend on the choice of $\gamma(\xi,\omega)$.

Finally, for $\nu_p = p_1\nu_1 + ... + p_k\nu_k$ and $c_p = c_1p_1 + ... + c_kp_k$, we get:

$$\begin{aligned}
W((1-p_0')\mu + &p_0'\xi, p_0\omega + \nu_p) + c_p = \\
&= \kappa p_0 + \kappa' p_0' + c^T p + \min_{g,g'} \int_{t\in\mathbb{R}} |(1-p_0')M(t) - G(t) - N(t) + G'(t)|\, dt.
\end{aligned}$$

The term $\kappa p_0 + \kappa' p_0' + c^T p$ does not depend neither on g nor g', so after including minimization

over p'_0, p_0, p we get:

$$\min_{p'_0,p_0,p}\Big(W((1-p'_0)\mu + p'_0\xi, p_0\omega + \nu_p) + c_p\Big) =$$
$$= \min_{p'_0,p_0,p,g,g'}\left(\kappa p_0 + \kappa' p'_0 + c^T p + \int_{t\in\mathbb{R}} |(1-p'_0)M(t) - G(t) - N(t) + G'(t)|dt\right),$$

where $\nu_p = p_1\nu_1 + \cdots + p_k\nu_k$, $c_p = c_1 p_1 + \cdots + c_k p_k$, $c^T p = c_1 p_1 + \ldots c_k p_k$. As we consider the discrete case the above equation is exactly our thesis. □

As seen from Theorem 2 instead of Eq. (1) we can consider:

$$p'^*_0, p^*_0, p^*, g^*, g'^* =$$
$$= \arg\min_{p'_0,p_0,p,g,g'} \{\kappa_0 p_0 + \kappa' p'_0 + c^T p + \sum_{i=1}^{n-1}(s_{i+1} - s_i)\,|(1-p'_0)M(s_i) + G'(s_i) - G(s_i) - N_p(s_i)|\}$$

as our minimization problem and use linear programming to solve it.

Theorem 3. *Problem*

$$p'^*_0, p^*_0, p^*, g^*, g'^* =$$
$$= \arg\min_{p'_0,p_0,p,g,g'} \{\kappa_0 p_0 + \kappa' p'_0 + c^T p + \sum_{i=1}^{n-1}(s_{i+1} - s_i)\,|(1-p'_0)M(s_i) + G'(s_i) - G(s_i) - N_p(s_i)|\}$$

is equivalent to

$$
\begin{aligned}
\textit{maximize} \quad & V^T\mathbf{z} \\
\textit{subject to} \quad & (V')^T\mathbf{z} \leq \kappa', \\
& W^T\mathbf{z} \leq -\kappa + C, \\
& z_i - z_n \leq 0 \quad \textit{for } i = 1, 2, ..., n-1, \\
& -z_i - z_{n+1} \leq 0 \quad \textit{for } i = 1, 2, ..., n-1, \\
& z_i - z_{i+1} \leq l_i \quad \textit{for } i = 1, 2, ..., n-2, \\
& z_{n-1} \leq l_{n-1}, \\
& z_i - z_{i+1} \geq -l_i \quad \textit{for } i = 1, 2, ..., n-2, \\
& z_{n-1} \geq -l_{n-1}, \\
& z_n, z_{n+1}, z_{n+2}, z_{n+3} \geq 0, \\
& \mathbf{z} \in \mathbb{R}^{n+3},
\end{aligned}
$$

where

$$C = \begin{bmatrix} c_1 & c_2 & \dots & c_k \end{bmatrix}^T$$

$$-\kappa + C = \begin{bmatrix} -\kappa + c_1 & -\kappa + c_2 & \dots & -\kappa + c_k \end{bmatrix}^T,$$

$$V := \begin{bmatrix} \mu(s_1) & \mu(s_2) & \dots & \mu(s_{n-1}) & -1 & 0 & 0 & -1 \end{bmatrix}^T,$$

$$V' := \begin{bmatrix} \mu(s_1) & \mu(s_2) & \dots & \mu(s_{n-1}) & 0 & 1 & -1 & 0 \end{bmatrix}^T,$$

$$W := \begin{bmatrix} \nu_1(s_1) & \nu_2(s_1) & \dots & \nu_k(s_1) \\ \nu_1(s_2) & \nu_2(s_2) & \dots & \nu_k(s_2) \\ \vdots & \vdots & \ddots & \vdots \\ \nu_1(s_{n-1}) & \nu_2(s_{n-1}) & \dots & \nu_k(s_{n-1}) \\ -1 & -1 & \dots & -1 \\ 0 & 0 & \dots & 0 \\ 1 & 1 & \dots & 1 \\ -1 & -1 & \dots & -1 \end{bmatrix}.$$

Proof of Theorem 3 is analogous to the proof of Theorem 3 from.[2] Below we prove our version of the Theorem with the extension necessary to incorporate penalties into a linear program.

Proof. Let's denote:

$$\begin{aligned} l_i &:= s_{i+1} - s_i, \\ M_i &:= M(s_i), \\ N_{ij} &:= N_j(s_i), \\ N_{ij} &:= N_j(s_i), \\ G_i &:= G(s_i), \\ G'_i &:= G'(s_i), \\ g_i &:= g(s_i), \\ g'_i &:= g'(s_i), \\ \epsilon_i &:= (1 - p'_0)M_i + G'_i - G_i - \sum_{j=1}^{k} p_j N_{ij}. \end{aligned}$$

Using this notation, we can rewrite our minimization problem as:

$$\begin{aligned}
\text{minimize} \quad & \sum_{i=1}^{n-1} l_i|\epsilon_i| + \kappa' p_0' + \kappa p_0 + \sum_{j=1}^{k} p_j c_j \quad \text{over } \epsilon, p_0', p_0, p, g, g' \\
\text{subject to} \quad & \epsilon_i + \sum_{j=1}^{i} g_j - \sum_{j=1}^{i} g_j' + \sum_{j=1}^{k} p_j N_{ij} + p_0' M_i = M_i \quad \text{for } i = 1, ...n-1, & (19) \\
& \sum_{i=1}^{n} g_j \leq p_0, & (20) \\
& \sum_{i=1}^{n} g_j' \leq p_0', & (21) \\
& 1 - p_0 - p_0' \geq 0, & (22) \\
& \sum_{j=1}^{k} p_j \leq 1, & (23) \\
& \epsilon_i^+, \epsilon_i^-, p_0', p_0, p_i, g_i, g_i' \geq 0
\end{aligned}$$

Constraint Eq. (19) is a consequence of the definition of ϵ_i, Eq. (20), and Eq. (21) follow from the fact that the total amount of signal removed from experimental and theoretical spectra cannot be greater than p_0 and p_0', respectively. Those constraints aren't equalities, as we assume that some amount of signal: $\gamma(\xi, \omega)$, might be transported from ξ to ω. This situation lacks clear interpretation and, as shown in[2] is never optimal, thus won't occur in practice. Moreover, constraint Eq. (22) is the same as assumption Eq. (3), and Eq. (23) states that the sum of estimated proportions must be less or equal to 1.

To obtain a linear program in a standard form, we need a linear function and non-negative variables. Thus, we split ϵ_i and $|\epsilon_i|$ using positive and negative parts:

$$\epsilon_i = \epsilon_i^+ - \epsilon_i^-,$$

$$|\epsilon_i| = \epsilon_i^+ + \epsilon_i^-.$$

We can also get rid of p_0 by substituting

$$p_0 = 1 - \sum_{j=1}^{k} p_j.$$

Consequently, we obtain the following formulation:

$$\begin{aligned}
\text{minimize} \quad & \sum_{i=1}^{n-1} l_i \epsilon_i^+ \sum_{i=1}^{n-1} l_i \epsilon_i^- + \kappa' p_0' + \sum_{j=1}^{k} (c_j - \kappa) p_j \quad \text{over } \epsilon^+, \epsilon^-, p_0', p_0, p, g, g' \\
\text{subject to} \quad & \epsilon_i^+ - \epsilon_i^- + \sum_{j=1}^{i} g_j - \sum_{j=1}^{i} g_j' + \sum_{j=1}^{k} p_j N_{ij} + p_0' M_i = M_i \quad \text{for } i = 1, ...n-1, \\
& - \sum_{i=1}^{n} g_j - \sum_{j=1}^{k} p_j \geq -1, \\
& p_0' - \sum_{i=1}^{n} g_j' \geq 0, \\
& \sum_{j=1}^{k} p_j - p_0' \geq 0, \\
& - \sum_{j=1}^{k} p_j \geq -1, \\
& \epsilon_i^+, \epsilon_i^-, p_0', p_0, p_j, g_i, g_i' \geq 0,
\end{aligned}$$

which is a linear program. Note that we skip a constant $+\kappa$ in the objective function as minimizing an expression plus constant is equivalent to minimizing an expression itself. To simplify the computations, we find a dual formulation of our minimization problem:

$$
\begin{aligned}
\text{maximize} \quad & \sum_{i=1}^{n-1} M_i x_i - x_n - x_{n+3} \quad \text{over } \mathbf{x} \\
\text{subject to} \quad & x_i \leq l_i \quad \text{for } i = 1, ..., n-1, && (24)\\
& -x_i a \leq l_1 \quad \text{for } i = 1, ..., n-1, && (25)\\
& \sum_{i=1}^{n-1} M_i x_i + x_{n+1} - x_{n+2} \leq \kappa', && (26)\\
& \sum_{i=1}^{n-1} N_{ij} x_i - x_n + x_{n+2} - x_{n+3} \leq -\kappa + c_j \quad \text{for } j = 1, ..., k, && (27)\\
& \sum_{k=i}^{n-1} x_k - x_n \leq 0 \quad \text{for } i = 1, ..., n-1, && (28)\\
& -x_n \leq 0, && (29)\\
& -\sum_{k=i}^{n-1} x_k - x_{n+1} \leq 0 \quad \text{for } i = 1, ..., n-1, && (30)\\
& -x_{n+1} \leq 0, && (31)\\
& x_n, x_{n+1}, x_{n+2}, x_{n+3} \geq 0 \\
& \mathbf{x} \in \mathbb{R}^{n+3}.
\end{aligned}
$$

Firstly, let's note that Eq. (29) and Eq. (31) are included in the non-negativity constraint, thus we will omit them in the future. Now we want to switch to matrix notation, so let's define the following vectors and matrices $L \in \mathbb{R}^{n-1}, C \in R^k, M \in \mathbb{R}^{n-1}, U \in M_{(n-1)\times(n-1)}, N \in M_{(n-1)\times k}, C \in M_{(n-1)\times k}, \widetilde{V} \in \mathbb{R}^{n-1}, \widetilde{W} \in M_{(n-1)\times k}$ as:

$$L := \begin{bmatrix} l_1 & l_2 & \dots & l_{n-1} \end{bmatrix}^T,$$

$$C := \begin{bmatrix} c_1 & c_2 & \dots & c_k \end{bmatrix}^T,$$

$$M := \begin{bmatrix} M_1 & M_2 & \dots & M_{n-1} \end{bmatrix}^T,$$

$$U := \begin{bmatrix} 1 & 0 & \dots & 0 \\ 1 & 1 & \dots & 0 \\ \vdots & \vdots & \ddots & \vdots \\ 1 & 1 & \dots & 1 \end{bmatrix},$$

$$N := \begin{bmatrix} N_{11} & N_{12} & \dots & N_{1k} \\ N_{21} & N_{22} & \dots & N_{2k} \\ \vdots & \vdots & \ddots & \vdots \\ N_{(n-1)1} & N_{(n-1)2} & \dots & N_{(n-1)k} \end{bmatrix},$$

$$\widetilde{V} := \begin{bmatrix} \mu(s_1) & \mu(s_2) & \dots & \mu(s_{n-1}) \end{bmatrix}^T,$$

$$\widetilde{W} := \begin{bmatrix} \nu_1(s_1) & \nu_2(s_1) & \dots & \nu_k(s_1) \\ \nu_1(s_2) & \nu_2(s_2) & \dots & \nu_k(s_2) \\ \vdots & \vdots & \ddots & \vdots \\ \nu_1(s_{n-1}) & \nu_2(s_{n-1}) & \dots & \nu_k(s_{n-1}) \end{bmatrix}.$$

Note that $M = U\widetilde{V}$ and $N = U\widetilde{W}$. Thus to make computations more efficient, we can use: $\widetilde{V}, \widetilde{W}$ rather than M, N, as they are sparse. Moreover, we define the addition of $-\kappa$ penalty and vector of penalties C as:

$$-\kappa + C = \begin{bmatrix} -\kappa + c_1 & -\kappa + c_2 & \dots & -\kappa + c_k \end{bmatrix}^T.$$

Moreover, for ease of notation let's split dual variables vector $\mathbf{x}$ into two vectors:

$$\widetilde{\mathbf{x}} = \begin{bmatrix} x_1 & x_2 & \dots & x_{n-1} \end{bmatrix}^T,$$
$$\widehat{\mathbf{x}} = \begin{bmatrix} x_n & x_{n+1} & x_{n+2} & x_{n+3} \end{bmatrix}^T.$$

Now we can rewrite the dual program using matrix notation as:

$$
\begin{aligned}
\text{maximize} \quad & \widetilde{V}^T U^T \widetilde{\mathbf{x}} + \begin{bmatrix} 1 & 0 & 0 & -1 \end{bmatrix} \widehat{\mathbf{x}} \\
\text{subject to} \quad & \widetilde{\mathbf{x}} \leq L, \\
& -\widetilde{\mathbf{x}} \leq L, \\
& \widetilde{V}^T U^T \widetilde{\mathbf{x}} + \begin{bmatrix} 0 & 1 & -1 & 0 \end{bmatrix} \widehat{\mathbf{x}} \leq \kappa' \\
& \widetilde{W}^T U^T \widetilde{\mathbf{x}} + \begin{bmatrix} -1 & 0 & 1 & -1 \\ -1 & 0 & 1 & -1 \\ \vdots & \vdots & \vdots & \vdots \\ -1 & 0 & 1 & -1 \end{bmatrix} \widehat{\mathbf{x}} \leq -\kappa + C, \\
& U^T \widetilde{\mathbf{x}} + \begin{bmatrix} -1 & 0 & 0 & 0 \\ -1 & 0 & 0 & 0 \\ \vdots & \vdots & \vdots & \vdots \\ -1 & 0 & 0 & 0 \end{bmatrix} \widehat{\mathbf{x}} \leq 0, \\
& -U^T \widetilde{\mathbf{x}} + \begin{bmatrix} 0 & -1 & 0 & 0 \\ 0 & -1 & 0 & 0 \\ \vdots & \vdots & \vdots & \vdots \\ 0 & -1 & 0 & 0 \end{bmatrix} \widehat{\mathbf{x}} \leq 0, \\
& \widehat{\mathbf{x}} \geq 0.
\end{aligned}
$$

To further simplify the computations we change variables:

$$\mathbf{z} := \begin{bmatrix} 1 & 1 & \dots & 1 & 0 & 0 & 0 & 0 \\ 0 & 1 & \dots & 1 & 0 & 0 & 0 & 0 \\ \vdots & \vdots & \ddots & \vdots & \vdots & \vdots & \vdots & \vdots \\ 0 & 0 & \dots & 1 & 0 & 0 & 0 & 0 \\ 0 & 0 & \dots & 0 & 1 & 0 & 0 & 0 \\ 0 & 0 & \dots & 0 & 0 & 1 & 0 & 0 \\ 0 & 0 & \dots & 0 & 0 & 0 & 1 & 0 \\ 0 & 0 & \dots & 0 & 0 & 0 & 0 & 1 \end{bmatrix} \mathbf{x}.$$

The above matrix has $n+3$ rows and $n+3$ columns, where the first $n-1$ rows and columns correspond to $U^T \in M_{(n-1)\times(n-1)}$ and the latter four to identity matrix. The other way of writing variable substitution would be:

$$\mathbf{z} := \begin{bmatrix} U^T & O_{(n-1)\times 4} \\ O_{4\times(n-1)} & I_4 \end{bmatrix} \mathbf{x},$$

where O is a zero matrix and I is identity matrix.

If we denote

$$\widetilde{\mathbf{z}} := \begin{bmatrix} z_1 & z_2 & \dots z_{n-1} \end{bmatrix}^T,$$
$$\widehat{\mathbf{z}} := \begin{bmatrix} z_n & z_{n+1} & z_{n+2} & z_{n+3} \end{bmatrix}^T,$$

then the following equalities hold:

$$\widetilde{\mathbf{z}} = U^T \widetilde{\mathbf{x}},$$
$$\widehat{\mathbf{z}} = \widehat{\mathbf{x}}.$$

Furthermore, since $(U^T)^{-1}$ exists and

$$(U^T)^{-1} = \begin{bmatrix} 1 & -1 & 0 & \dots & 0 & 0 \\ 0 & 1 & -1 & \dots & 0 & 0 \\ \vdots & \vdots & \vdots & \ddots & \vdots & \vdots \\ 0 & 0 & 0 & \dots & 1 & -1 \\ 0 & 0 & 0 & \dots & 0 & 1 \end{bmatrix},$$

the following also holds:

$$x_i = z_i - z_{i+1} \quad \text{for } i = 1, ..., n-2,$$

$$x_{n-1} = z_{n-1}.$$

Using the substitution and equalities described above, we can rewrite our optimization prob-

lem as:

$$
\begin{aligned}
\text{maximize} \quad & \widetilde{V}^T \widetilde{\mathbf{z}} + \begin{bmatrix} -1 & 0 & 0 & -1 \end{bmatrix} \widehat{\mathbf{z}} \\
\text{subject to} \quad & z_i - z_{i+1} \leq l_i \quad \text{for } i = 1, ..., n-2, \\
& z_{n-1} \leq l_{n-1}, \\
& z_i - z_{i+1} \geq -l_i \quad \text{for } i = 1, ..., n-2, \\
& z_{n-1} \geq -l_{n-1}, \\
& \widetilde{V}^T \widetilde{\mathbf{z}} + \begin{bmatrix} 0 & 1 & -1 & 0 \end{bmatrix} \widehat{\mathbf{z}} \leq \kappa' \\
& \widetilde{W}^T \widetilde{\mathbf{z}} + \begin{bmatrix} -1 & 0 & 1 & -1 \\ -1 & 0 & 1 & -1 \\ \vdots & \vdots & \vdots & \vdots \\ -1 & 0 & 1 & -1 \end{bmatrix} \widehat{\mathbf{z}} \leq -\kappa + C, \\
& \widetilde{\mathbf{z}} + \begin{bmatrix} -1 & 0 & 0 & 0 \\ -1 & 0 & 0 & 0 \\ \vdots & \vdots & \vdots & \vdots \\ -1 & 0 & 0 & 0 \end{bmatrix} \widehat{\mathbf{z}} \leq 0, \\
& \widetilde{\mathbf{z}} + \begin{bmatrix} 0 & -1 & 0 & 0 \\ 0 & -1 & 0 & 0 \\ \vdots & \vdots & \vdots & \vdots \\ 0 & -1 & 0 & 0 \end{bmatrix} \widehat{\mathbf{z}} \leq 0 \\
& z_n, z_{n+1}, z_{n+2}, z_{n+3} \geq 0, \\
& \widetilde{\mathbf{z}} \in \mathbb{R}^{n-1}, \widehat{\mathbf{z}} \in \mathbb{R}^4.
\end{aligned}
$$

Now, if we define $V, V' \in \mathbb{R}^{n+3}, W \in M_{(n+3)\times(n+3)}$ as:

$$
V := \begin{bmatrix} \mu(s_1) & \mu(s_2) & \dots & \mu(s_{n-1}) & -1 & 0 & 0 & -1 \end{bmatrix}^T,
$$

$$V' := \begin{bmatrix} \mu(s_1) & \mu(s_2) & \dots & \mu(s_{n-1}) & 0 & 1 & -1 & 0 \end{bmatrix}^T,$$

$$W := \begin{bmatrix} \nu_1(s_1) & \nu_2(s_1) & \dots & \nu_k(s_1) \\ \nu_1(s_2) & \nu_2(s_2) & \dots & \nu_k(s_2) \\ \vdots & \vdots & \ddots & \vdots \\ \nu_1(s_{n-1}) & \nu_2(s_{n-1}) & \dots & \nu_k(s_{n-1}) \\ -1 & -1 & \dots & -1 \\ 0 & 0 & \dots & 0 \\ 1 & 1 & \dots & 1 \\ -1 & -1 & \dots & -1 \end{bmatrix},$$

then we get the final formulation of our optimization problem, that is:

$$\begin{aligned} \text{maximize} \quad & V^T\mathbf{z} \quad \text{over } \mathbf{z} \\ \text{subject to} \quad & (V')^T\mathbf{z} \le \kappa', \\ & W^T\mathbf{z} \le -\kappa + C, \\ & z_i - z_n \le 0 \quad \textit{for } i = 1, 2, ..., n-1, \\ & -z_i - z_{n+1} \le 0 \quad \textit{for } i = 1, 2, ..., n-1, \\ & z_i - z_{i+1} \le l_i \quad \textit{for } i = 1, 2, ..., n-2, \\ & z_{n-1} \le l_{n-1}, \\ & z_i - z_{i+1} \ge -l_i \quad \textit{for } i = 1, 2, ..., n-2, \\ & z_{n-1} \ge -l_{n-1}, \\ & z_n, z_{n+1}, z_{n+2}, z_{n+3} \ge 0, \\ & \mathbf{z} \in \mathbb{R}^{n+3}. \end{aligned}$$

□

References

(1) Santambrogio, F. *Optimal Transport for Applied Mathematicians: Calculus of Variations, PDEs, and Modeling*; Progress in Nonlinear Differential Equations and Their Applications; Springer International Publishing: Cham, 2015; Vol. 87.

(2) Domżał, B.; Nawrocka, E. K.; Gołowicz, D.; Ciach, M. A.; Miasojedow, B.; Kazimierczuk, K.; Gambin, A. Magnetstein: An Open-Source Tool for Quantitative NMR Mixture Analysis Robust to Low Resolution, Distorted Lineshapes, and Peak Shifts. *Analytical Chemistry* **2023**, *96*, 188–196.
